# The mEPN scheme: an intuitive and flexible graphical system for rendering biological pathways

**DOI:** 10.1186/1752-0509-4-65

**Published:** 2010-05-17

**Authors:** Tom C Freeman, Sobia Raza, Athanasios Theocharidis, Peter Ghazal

**Affiliations:** 1 Division of Pathway Medicine, University of Edinburgh Medical School, The Chancellor's Building, College of Medicine, 49 Little France Crescent, Edinburgh, EH16 4SB, UK; 2 The Roslin Institute and Royal (Dick) School of Veterinary Studies, University of Edinburgh, Roslin, Midlothian, EH25 9PS, UK; 3 Centre for Systems Biology at Edinburgh, C H Waddington Building, King's Buildings, Mayfield Road, Edinburgh, EH9 3JU, UK

## Abstract

**Background:**

There is general agreement amongst biologists about the need for good pathway diagrams and a need to formalize the way biological pathways are depicted. However, implementing and agreeing how best to do this is currently the subject of some debate.

**Results:**

The modified Edinburgh Pathway Notation (mEPN) scheme is founded on a notation system originally devised a number of years ago and through use has now been refined extensively. This process has been primarily driven by the author's attempts to produce process diagrams for a diverse range of biological pathways, particularly with respect to immune signaling in mammals. Here we provide a specification of the mEPN notation, its symbols, rules for its use and a comparison to the proposed Systems Biology Graphical Notation (SBGN) scheme.

**Conclusions:**

We hope this work will contribute to the on-going community effort to develop a standard for depicting pathways and will provide a coherent guide to those planning to construct pathway diagrams of their biological systems of interest.

## Background

Pathway diagrams are currently available in a plethora of different forms. Using the term in the broadest sense, they can be a picture that accompanies a review article, wall charts distributed by journals and companies, small schematic diagrams used to support mathematical modeling efforts or network graphs reflecting known protein interactions based on the results of large scale interaction studies or automated literature mining. To support these efforts there are also a growing number of databases that serve up these 'pathways' [1]. These are either curated centrally [2-5] or increasingly by the community [6-8]. The sheer range of resources available reflects the current interest in pathway science. However, this variety can in itself be frustrating. Pathways are drawn using informal and idiosyncratic notation systems, with varying degrees of accuracy and specificity in defining what pathway components are being depicted and the relationships between them. Resources are often fragmented with some proteins or metabolites being members of numerous pathways; the concept of pathway membership being a highly subjective division. The pathways themselves are rarely available as a cohesive network and there are numerous pathway exchange formats in use. All in all, despite the huge efforts in time and resources that has been poured into pathway science the state of the art leaves a lot to be desired. The advent of analytical techniques able to perform genome-wide analysis of cell systems has opened a window to our comprehension of systems-level biology. It has however also highlighted the pressing need for comprehensive pathway models in order to assist with the interpretation of this data.

In recognition of these issues a number of groups have proposed formalized notation schemes for drawing 'wiring diagrams' of cellular pathways [9-12]. The process diagram notation (PDN) on which our work has been largely based [9], has been used in the generation of a number of relatively large pathway diagrams [13-15]. However, in the course of our investigations we have found that the diagrams resulting from these elegant and pioneering efforts were not always easy to interpret and the notation system was a challenge to implement. Furthermore, we found that the PDN did not support all of the concepts that are required to reflect the diversity of pathway components and the relationships between them. The original Edinburgh Pathway Notation (EPN) scheme [11] was designed to allow the logical depiction of signaling pathways. The basic objectives of the EPN were to create a notation scheme that was: a) flexible enough to allow the detailed representation of a diverse range of biological entities, interactions and pathway concepts; b) able to represent pathway knowledge in a semantically and visually unambiguous manner; c) able to the construct pathway diagrams that are understandable by a biologist; and d) able to produce diagrams that are sufficiently well defined that software tools can convert graphical models into formal models suitable for analysis and simulation. It incorporated many of the ideas of the process PDN scheme but notably introduced the idea of using Boolean logic operators (AND/OR/NOT) nodes to represent co-dependencies between components. As our pathway mapping efforts have continued to develop and been driven by our interest in modeling a diverse range of biological pathways and concepts, we found it necessary to further refine the EPN scheme. We are now satisfied that this graphical language has reached a sufficient level of maturity to now formally describe the 'modified' EPN scheme. In doing so we seek to provide a cohesive guide for those wanting to construct any range of pathways using the mEPN, and support our own work in depicting the regulation of macrophage biology [16-18]. We also believe that the mEPN scheme has some important advantages over other proposed pathway notation schemes and is therefore a positive contribution to the debate on standardizing pathway depiction.

## Results

### Definition of the modified Edinburgh Pathway Notation (mEPN) Scheme

**Figure 1:**
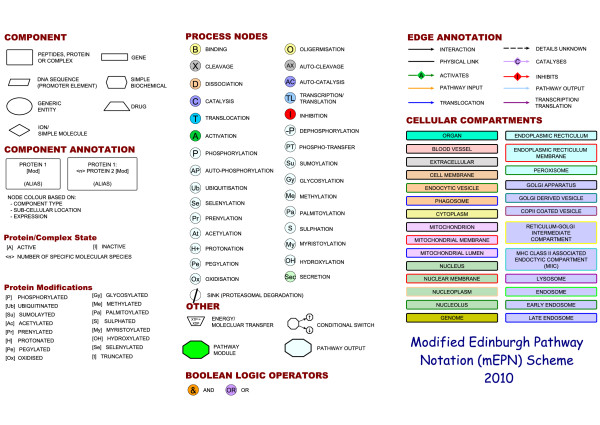
**List of the Glyphs used by the modified Edinburgh Pathway Notation (mEPN) scheme**. Unique shapes and identifiers are used to distinguish between each element of the notation scheme. The notation scheme essentially consists of the following categories of nodes representing; cellular components, compartments, Boolean logic, edge annotations, reactions and processes. For a full description of the notation scheme and rules for its use see Additional files 1 and 2.

A *pathway *may be considered to be a directional network of molecular *interactions *between *components *of a biological system that act together to regulate a cellular event or process. In this context a *component *is any physical entity involved in a pathway e.g. a protein, protein complex, nucleic acid (DNA, RNA), molecule, etc. *Interactions *are generally the relationships between one component and another where one component influences the activity of another e.g. through its binding to, inhibition of, catalytic conversion of, etc. Interactions between cellular components thereby lead to a change in the status of the system. A *pathway notation scheme *is a collection of predefined symbols (shapes, lines, figures) that represent the constituent parts of a graphical system for depicting the components of a biological pathway, the interactions between them and the cellular compartments in which they occur. A scheme should also include rules for the use of these symbols in depicting information. *Glyphs *are stylized graphical symbols that impart information nonverbally and are used to portray different classes of biological entities e.g. protein, gene, pathogen etc. and the nature of the relationships between them. In network terminology all glyphs are nodes (vertices) of a specific type and the connectivity between them is defined by *edges *(lines/arcs). The full set of glyphs employed in the mEPN scheme are shown in figure 1 and a full description of them and their use is given in the accompanying mEPN specification document (Additional files 1 and 2).

### Pathway Components

The mEPN uses a set of standard shapes to represent classes of molecules (components) from a rounded rectangle to represent proteins and protein complexes, to a diamond shaped glyph to represent simple ions and molecules e.g. Na^+^, K^+^, H_2_O etc. Components play some role within the pathway and exist in one or a number of locations within a cell. An important rule of the mEPN is that a component may only be represented once in any given cellular compartment. Whilst this rule can potentially lead to a tangle of edges due to certain components possessing numerous connections to other components spread across the pathway, the benefits of the rule outweigh the issues in adhering to it. The number of edges entering or leaving each node gives the reader an exact indication of a component's connections to other components and hence potential activity, without the need for scanning the entire diagram to find other instances where the component is described. A notable exception to this rule is in the depiction of small and ubiquitously present ions and molecules which may be represented numerous times and be involved in numerous processes. A component may however be shown more than once in a given cellular compartment if it changes from one state to another e.g. from an inactive form to an active form, in which case both forms are represented as separate components.

**Figure 2:**
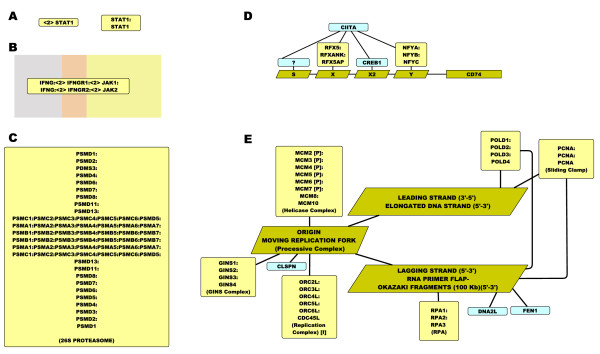
**Graphical Representation of Complexes**. **(A) **Two alternate views of the STAT1 homodimer both of which would be considered to be formally correct under the mEPN scheme. **(B) **Visual representation of interferon-gamma receptor complex bound to IFNG. For membrane receptor complexes such this we have generally favoured showing the complexes spanning the plasma membrane (brown) with the receptor portion protruding into the extra-cellular space (grey) and with the adaptor molecules projecting into the cytoplasm (yellow). **(C) **The 26S proteasome has a barrel-like structure made up of 6 six concentric rings each composed of 7 proteasome subunits, capped with regulatory subunits at either end. We therefore chose to arrange the subunit in names in this manner so as to capture visually something of this arrangement. **(D) **Model of the transcription factor/coactivator complex that regulate genes associated with MHC class2 antigen presentation such as *CD74*. In this case transcription is thought to be regulated by two transcription factor complexes, CREB1 and one unknown factor which bind directly to four elements in the gene's promoter and transcription is initiated by a conformation change induced by the binding of CIITA. Here as in E interaction edges are used to denote a physical link between components of the complex. **(E) **DNA replication complex formed during S-phase. As complexes become large the use of the physical interaction edge becomes essential in defining not only which components make up the complex but where in the complex they reside. This arrangement also allows for the depiction of specific components of the complex to undergo a change in state or cause a change in another component (which may or may not be part of the same complex).

It is worth considering the depiction of protein complexes as a special case. For relatively simple protein complexes e.g. dimers, trimers, it is usually sufficient to depict the complex as a simple rounded rectangle labelled using the names of the constituent proteins and their modifications (Figure 2a). However as the size of complexes grows this can become limiting. In the first instance a complex may span more than one cellular compartment e.g. a membrane receptor complex and it is useful to depict this and the relative position of molecules within the complex. In such cases we have found it useful to draw the complex as an elongated rounded rectangle with the names of the proteins arranged relative to their position within the complex/cell (Figure 2b). This also has the added advantage of reducing the reducing space a complex takes up, a distinct benefit when depicting a large number of receptors and their transient activation states. In other cases a protein complex may have a well recognised structure and it may be desirable to reflect that structure in its depiction. For example when depicting various forms of the proteasome we arranged the subunit names in layers reflecting the composition of the proteasome's core barrel structure and placed the cap-proteins at either end of this barrel. Whilst far from perfect it goes some way in capturing the recognised structure of the complex. However at a point this too becomes limiting and when components are complexes of complexes or complexes composed of proteins and say DNA, it is no longer sufficient to simply represent everything as single unified component. In such cases we have found it useful to depict complexes as a collection of components joined using non-directional edges (an edge without an arrowhead) which represent a physical covalent of non-covalent bond. In this way a functional entity can be seen to be composed of multiple separate entities each of which can be separately modified but still influence the activity and composition as a whole (Figure 2c).

### Component annotation

Multiple names are often available to describe any given protein with a number of different protein names frequently in use in the literature at any one time. Likewise some common names may be used to describe more than one protein or complex. This use of non-standard nomenclature frequently leads to ambiguity as to the exact identity of the component being depicted. Under mEPN we therefore recommend the use of standard gene nomenclature systems e.g. HGNC or MGD to name human or mouse genes/proteins, respectively. These nomenclature systems now provide a near complete annotation of all human and mouse genes and their use in the naming of proteins provides a direct visual link between the identity of the gene and the corresponding protein. Where other names (alias') are in common use these names may be shown as an addition to the label on the glyph representing the protein and are included as part of the node's label after the official gene symbol in rounded () brackets. Use of standard nomenclature also assists in the comparison and overlay of experimental data (which is usually annotated using standard nomenclature) with pathway models. At the present time there are unfortunately no standard and universally recognized nomenclature systems available for naming certain types of pathway components. For instance protein isoforms tend to be named in an ad hoc manner by those who study them and biochemical compounds are known by both their common names or by names that reflect their chemical composition. For instance the IUPAC nomenclature system http://www.chem.qmul.ac.uk/iupac/ is a standard nomenclature system for organic chemicals but most names would have little relevance to a biologist. In cases such as these the important thing is to be consistent and where possible to cross reference the components ID to other sources such that the identity of the component depicted, where at all possible, is unambiguous.

Protein complexes when drawn as a single entity are named as a concatenation of the proteins belonging to the complex separated by a colon. Again if the complex is commonly referred to by a generic name this may be shown below the constituent parts. There are no strict rules as to the order in which the protein names are shown in the complex and are often shown in the order in which proteins join the complex, in the position they are likely to hold relative to other members of the complex (where known) or position relative to cellular compartments e.g. with receptor proteins in a membrane bound protein complex protruding into the extra-cellular space. Where a specific protein is present multiple times within a complex, this may be represented by placing the number of times a protein is present within the complex in angle brackets < >. If the number of proteins in the complex is unknown this may be represented by <n>. The particular 'state' of an individual protein or a protein within a complex may be altered as a consequence of a particular process. This change in the component's state is marked using square [] brackets following the component's name; each modification being placed in separate brackets. This notation may be used to describe the whole range of protein modifications from phosphorylation [P], truncation [t], ubquitinisation [Ub] etc. Where details of the site of modification are known this may be represented e.g. [P-L232] = phosphorylation at leucine 232. Alternatively the details of a particular modification may be placed as a note on the node visible only during 'mouse-over' or when viewing a node's properties. Where multiple sites are modified this may be shown using multiple brackets, each modification (state) being shown in separate brackets.

### Depiction of Interactions between Components

Interactions are depicted by edges and signify the relationships between one component and another. Edges denote that an interaction occurs between components/processes in a pathway and convey the directionality of that interaction, where appropriate. The nature of an interaction is inferred through the use of edge annotation nodes, process nodes, and Boolean logic operators (see below). Interaction edges may be coloured for visual emphasis but as with nodes, the definition of meaning is not reliant on colour. A number of edges contain an in-line annotation node to indicate the 'type' of interaction, as is often depicted by the use of different arrowheads. An edge annotation is generally characterized as having only one input and one output, and functions to describe the type of activity implied by the edge e.g. activation, inhibition, catalysis. However, in certain instances they can be used as distribution nodes e.g. where one component activates many others such as with transcriptional activation of a number of genes by a transcription factor it can reduce the number of edges emanating from the transcription factor and therefore simply the representation. One other type of edge, one that connects components but has no arrowhead, is used to depict a physical interaction between the components. This can be used in the depiction of a bond between separate components of a complex, thereby providing improved visual clarity, especially with very large complexes, as to which components directly interact with each other.

### Depiction of Biological Processes

A process is a defined event occurring between components or to a component. A process node in the context of this notation system can be defined as a node that infers an action, transformation, transition or process. They impart information on the type of process that is associated with transformation of a component from one state to another or movement in cellular location. They also act as junctions between components and as such may have multiple inputs or outputs to components. In the mEPN all process nodes are represented by a small circular glyph and the process they represent is defined by a one-to-three letter code. Colour is used as a visual clue for quick recognition of the nature of the process depicted and group processes into 'type' but again is not necessary for inferring meaning. There are currently 31 process nodes recorded under the mEPN. Different process nodes generally have different connections. For instance a 'binding' node will have multiple inputs and one output, the opposite is true for a dissociation node. Process nodes also act as way of collating information about a given event; for example protein A may be cleaved by protein B, this reaction being ATP dependent. In this case A would be shown connected to its truncated form (A [t]) via a process node depicting cleavage (X). B would be shown to catalyze or active that process through its connection to the X-process node which would also receive an input from an energy transfer node (ATP->ADP) (See Additional file 3).

### Boolean Logic Operators

Boolean logic operators define the dependencies between components of a system describing the relationship between multiple inputs into a process. An 'AND' operator is used when two or more components are required to bring about a process i.e. an event is dependent on more than one factor being present. In modelling flow through networks these act in a similar manner to 'bind' process nodes i.e. all inputs must be present before a product is formed or reaction proceeds. In contrast an 'OR' operator is used when one component or another may orchestrate the same change in another component. For instance multiple kinases e.g. MAP2K3, MAP2K6, MAP2K7 may catalyze the phosphorylation of p38 (MAPK14) and therefore shown connecting with p38 via an OR operator. OR operators have also occasionally been used to infer that a component(s) has potentially multiple out comes.

### Other Nodes

There are a number of glyphs that represent concepts that do not sit neatly under the headings of being a component, a process or logic operator. These include:

*Energy/molecular transfer nodes *are used to represent simple co-reactions associated with or required to drive certain processes (e.g. ATP → ADP, GTP → GDP, NADPH → NADP^+^). They are linked directly to the node representing the process in which they take part.

*Conditional gates *are used where there are potentially multiple fates of a component and the output is dependant on other factors such as the components concentration, time or is associated with a cellular state. These have been used to depict control points such as the check point controls in cell cycle where the decision to go on to the next phase cell replication is under the control of a number of factors and two or more outcomes are possible. Another example is where cholesterol, depending on its intracellular concentration, may be either exported out of the cell or trigger the cholesterol synthesis pathway.

*Pathway modules *define complicated processes or events that are not otherwise fully described. Examples include signaling cascades, endocytosis, compartment fusion etc. They are a short-hand way of representing molecular events that are not known, not recorded or not shown.

*Pathway outputs *detail the cumulative output of series of interactions or function of an individual component at the 'end' of a pathway. Pathway outputs are shown in order to describe the significance of those interactions in the context of a biological process or with respect to the cell. The input lines leading into a pathway output node have been coloured light blue to emphasize the end of the pathway description.

### Compartments

A cellular compartment can be a region of the cell, an organelle or cellular structure, dedicated to particular processes and/or hosting certain sub-sets of components e.g. genes are found only in the nuclear compartment. Sub-cellular compartments are defined by a labelled background to the pathway and arranged with spatial reference to cell structure. Compartments are coloured differently for emphasis and to ease awareness the location of components. A proposed colour scheme for compartments is shown in Figure 1. Similar or related compartments share the same fill colour but have different coloured perimeters to define internal boundaries within a compartment e.g. membrane vs. lumen or to define the origin of compartments e.g. different classes of vesicles derived from the endoplasmic reticulum or plasma membrane.

### IFNγ Activation of MHC class II Gene Expression: A Worked Example of the mEPN in Use

**Figure 3:**
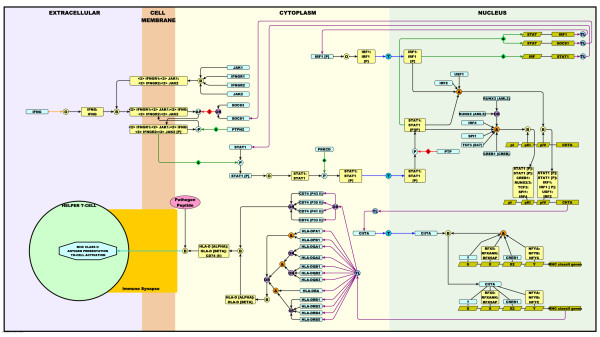
**Graphical Representation of the Interferon-gamma Pathway Leading to MHC class 2 Antigen Presentation**. Shown here are the known events between the release of IFNγ and the subsequent up-regulation of MHC class 2 antigen presentation by macrophages using the mEPN scheme. See results for a full description of this pathway.

In order to demonstrate the pathway notation system in action on a scale that can be viewed in this format, we have extracted a small section of our efforts in depicting macrophage biology [17,18]. Figure 3 depicts the activation of MHC class II genes by interferon-gamma (IFNG) as described in the literature and represented here using the mEPN scheme. Going through these series of events in detail:

IFNγ is secreted by T and NK cells upon activation [19-21] (not shown). It oligomerises to form a homodimer which then binds of to its receptor complex situated in the plasma membrane of macrophages [22]. This complex is formed from IFNGR1, IFNGR2, JAK1 and JAK2 [23,24], two copies of all proteins being present in the receptor complex. Binding of IFNγ causes the autophosphorylation of JAK2 [25] which in turn phosphorylates STAT1 [23]. The autophosphorylation of JAK2 can be inhibited by SOCS1 or SOCS3 [26,27], and the activated complex dephosphorylated by PTPN2 [28,29]. STAT1 now activated, oligomerises, is further phosphorylated by PRKCD [30] and translocates to the nucleus where it directly activates gene expression by binding to STAT sites present in the promoters of numerous genes. Shown on the diagram are just two of these genes, *SOCS1 *and *IRF1 *[31,32]. These form feedback inhibition and feed-forward activation loops, respectively. SOCS1 blocking further signal propagation through the inhibition of the IFNγ receptor complex (reviewed in [33] and IRF1 being necessary for the activation of *STAT1 *expression as well as being a necessary component of the *CIITA *transcriptional initiation complex [34]. At least two complexes are reported to be necessary to activate the expression of *CIITA *(reviewed in [35], the first composed of STAT1, IRF1, USF1 and IRF2 which binds to the so called pIV element of the *CIITA*, the second is comprised of STAT1, CREB1, RUNX2/3, TCF3, SPI1 and IRF4 which binds to the pIII element of the gene. CIITA is a co-activator and the key missing element in the transcription of MHC class II genes. Once translated it binds to a preassembled transcription factor complex, including members of the RFX and NFY family of proteins and CREB1, thereby activating the expression of the MHC class II genes [35]. This class of genes includes *CD74, HLA-DPA/B, HLA-DQA/B, HLA-DRA/B *[33,36] and through combinatorial assembly form a wide variety of complexes denoted here generically as CD74 (li):HLA-D (alpha):HLA-D (beta). It is this class of complexes that is shown in the main diagram to go on through a long series of steps to bind peptide antigen derived from the lysosomal degradation of pathogen proteins and present them to T-helper cells. As such this diagram serves as a graphical representation of the known pathway connecting IFNγ secretion to the activation of MHC class II antigen presentation.

Our work developing this notation scheme has reached a point where we foresee little need to change the majority of the mEPN scheme as presented here. Clearly the modeling of other systems and ideas from others however may in the future present a case for further modifications or refinements.

### Visualization of Pathway Information in 3D Environments

**Figure 4:**
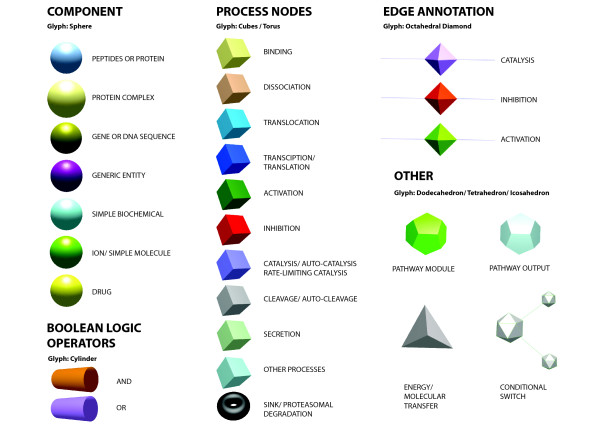
**mEPN^3D ^Scheme**. Presented here is a conversion of the standard mEPN scheme into a series of shapes that can be used to depict the same pathway concepts in 3D environments.

The reliance of the mEPN scheme on the principles of network graphs and use of simple node shapes, labels, edges and colour to convey pathway information has presented us with the opportunity to examine the use of other environments in which to visualize pathways. Layout of pathways in 3D space begins to address the issue of scalability associated with visualizing very large pathway diagrams and offers a little explored environment to visualize and interact with pathway models. Here we present for the first time a 3D translation of mEPN scheme (Figure 4). The scheme is devised to reflect the colours and where possible glyphs used in the 2D mEPN process diagrams converting the 2D shapes into 3D objects. The proposed notation mEPN^3D ^scheme is currently supported by the network visualization and analysis tool BioLayout *Express*^3D ^[37,38]http://www.biolayout.org/. This tool now supports the direct import of pathways as .graphml files, the main file type used by us to support our pathway modeling efforts. The potential of representing pathways in 3D environments is discussed below and elsewhere [16,17].

## Discussion

Pathway diagrams act as a visual representation of known portions of the vast molecular network that underpins all aspects of biological function. Models of pathways produced either as a graphical representation of known events or as a resource for mathematical modelling, are fundamental to understanding the workings of biological systems. However the task of assimilating the large amounts of available data and representing this information in an intuitive manner remains a challenge. Accordingly there has been increasing interest in the biology community to develop approaches for representing biological pathways. The Molecular Interaction Map (MIM) and Process Description Notation schemes were proposed by Kurt Kohn [10,39] and Hiroaki Kitano (Kitano 2005), respectively, and their ideas laid the foundations for much of the work on pathway notation that has followed. The current mEPN scheme is the based on ideas from the PDN and original EPN schemes but importantly the experience of over four years of pathway construction, notation testing and discussions.

The objectives of the EPN as originally proposed remain preserved, as do many of the original concepts of the EPN and PDN schemes [9,11]. However substantial modifications have been made to the notation system from the introduction of new symbols to changes in the aesthetics of the scheme and pathway syntax in order to achieve our original objectives. Firstly, we wanted a notation system that was flexible enough to allow the detailed representation of diverse biological entities, interactions and pathway concepts. In this respect, we have used the mEPN as described here not only in the construction of the large macrophage pathway diagrams [16-18] which in their own right cover a diverse range of signalling and effector pathways, but also for the depiction of cholesterol metabolism and the cell cycle (not shown). In all of these endeavours the mEPN scheme has been able to depict the literature-based understanding of these systems and where it was formerly unable to support a concept, it was modified to allow us to do so. Secondly, we wanted a system for presenting pathway knowledge in a semantically and visually unambiguous manner. To some degree this is down to actually labelling components in a way that is unambiguous. The use of standard gene nomenclature to label genes/protein components, together with a formalized system to describe modifications to them, goes someway to achieving this. This has meant in many cases that we have needed to first deconvolute the literature which describes these systems using numerous different names for the same protein or complex. It means however that one component is unlikely ever to be represented more than once but with different names. It also facilitates use of the diagrams in the interpretation of experimentally derived data which is frequently annotated using standard gene nomenclature. Our third aim, which is related to the second, is that diagrams are as simple as possible to construct and are understandable by a biologist. To help ensure this to be the case all the work in creating our pathway diagrams has been performed by relatively junior biologists (MSc/PhD students). They have been encouraged to discuss their ideas and their pathways with each other so as iron out areas where the information is not clearly depicted. For this to happen they must be able to communicate complicated biological concepts using the diagrams. The readability of a diagram is not only dependent on the notation system but also on its layout. Although a variety of automated layout algorithms exist for network graphs they do not perform as well as a human curator with an artistic eye for the task. Pathway layout is relatively trivial for small diagrams, but a long time has had to be spent on optimizing the layout all of our large pathways so that they are relatively easy to interpret. However, large integrated pathway diagrams, like the systems they represent, are inevitably complex. Finally, pathway diagrams are central to efforts to computationally model the observed behaviour of biological systems [40]. Our fourth objective has therefore been to develop the mEPN such that the semantics of the resulting network diagrams are sufficiently well defined that software tools can convert graphical models into formal models, suitable for analysis and simulation. Whilst the primary objective behind our efforts has been to create a graphical model of events, we have been mindful to construct pathway diagrams as directional networks that could in principle support studies on the dynamics of these systems. In examining various approaches to pathway modelling some are clearly not scalable, such as those using ordinary differential equations (ODEs) that require interaction parameters to be known or computed. Other approaches do not support the modelling of the co-dependencies between components of a pathway or give quantitative outputs (reviewed in [36,41]. However the recently published signaling Petri net (SPN) [42] potentially allows us to use diagrams constructed using the mEPN scheme to study the 'flow' of information through pathways. The SPN algorithm uses stochastic flow simulations to distribute 'tokens' representing quantitative estimates of activity through a network graph over time using only the network structure to determine outcomes. The technique has the advantage of offering fast computational simulations on large networks (< 1 sec for ~100 node networks), can support concepts of co-dependency between components and requires no kinetic details for interactions. In this way it should be possible to estimate the dynamics of information flow through a network and the effects of perturbations on that flow. Pathways drawn using the mEPN system can easily be converted into a bipartite graph of places (nodes) and transitions connected by arcs (edges) that are required to support this approach. We are currently exploring how SPN modelling might be used to better understand the structure and activity of the signalling systems of interest to us.

**Figure 5:**
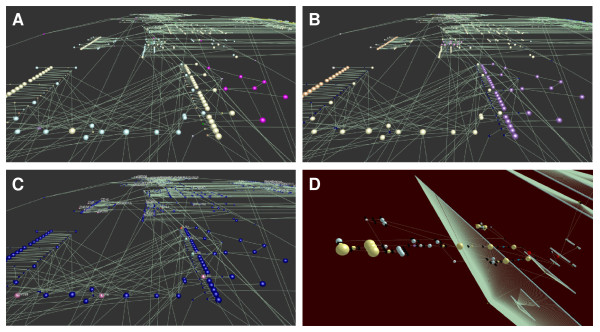
**Pathway Representation in 3D Environment**. Large macrophage activation pathway rendered in 3D environment where node shape, size and colour represents a components identity. **(A) **Nodes coloured according to type e.g. light blue - proteins, yellow - protein complexes, purple - generic molecular species. All process nodes are depicted as small cubes and coloured according to type. **(B) **Nodes coloured according to cellular location e.g. brown - plasma membrane, yellow - cytoplasm, purple - endosome, green nucleus. Process nodes/Boolean logic operators are shown as having no cellular location and are coloured dark blue (no class). **(C) **Nodes coloured according to overlay of data, in this case expression data. Colour of nodes represents co-expression cluster following stimulation of mouse macrophages with Ifnβ **(D) **A representation of the interferon-beta signalling pathway and the transcriptional network it controls. The signalling network is represented using the mEPN^3D ^notation with the addition of transition nodes for use in modelling studies. Connected to it are clusters of genes up or down regulated by Ifnb which have been stacked in at different layers depending on the their time course of activation/repression.

One advantage of the simple node and edge based approach to pathway element depiction is that it facilitates mEPN's conversion into other software environments. Graphml files (the main file exchange format used by the yEd editor) are supported by other network programs such as NodeXL http://www.codeplex.com/NodeXL, Sonivis http://www.sonivis.org/, GUESS http://guess.wikispot.org/GraphML, Pajek http://pajek.imfm.si/doku.php and NetworkX http://networkx.lanl.gov/ and the use of standard shaped nodes (glyphs) means that other generic network analysis tools such as Cytoscape [44] could also be used to draw mEPN diagrams. In particular we have been developing mEPN's compatibility with BioLayout *Express*^3D^, a network analysis tool developed by us for the visualization and analysis networks derived from 'omics data [37,38]. We have recently implemented a parser that supports the import of .graphml files into BioLayout *Express*^3D^. This translates the visual characteristics and layout as defined by the original .graphml 2D node co-ordinates of mEPN pathway diagrams from yEd in to a series of 3D objects, each representing a different class component using a combination of shape, size and colour (Figure 4). Translating a 2D pathway into a 3D environment arguably offers no advantage for small diagrams. Indeed in 3D, arrowheads and polylines are not currently supported. However, when diagrams become large, pathways be rotated and viewed from any angle, zoomed in on and generally manipulated in an environment which is quite different to that of any 2D representation. In the 3D environment colour is a powerful device that can be used to further overlay visual information on to nodes (Figures 5a, b). Indeed we have now built in the ability of BioLayout *Express*^3D ^to directly export the analyses of one graph e.g. clusters from expression data and import and overlay this information on to another, in this instance a pathway (Figure 5c). It is also possible to imagine much larger models of pathway systems where the spatial layout of components in 3D space is based on a components cellular location (Figure 5d). With BioLayout *Express*^3D ^now capable of supporting networks comprising of up to 30,000-40,000 node graphs there is considerable scope for building ever larger pathway models and further exploring the potential of 3D environments for pathway visualization and analysis. One final use of the 3D environment is as a means to visualize pathway activity. We are now working on a version of BioLayout *Express*^3D ^that will harness the power of the OpenGL 3D graphics to animate analyses of flow through a pathway, again using a node's shape, size and colour to indicate a components activity during dynamic simulations of pathway activity.

**Figure 6:**
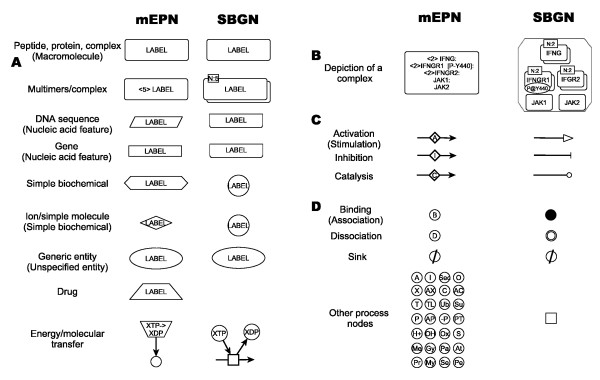
**Comparison of mEPN to SBGN**. Main glyphs used in the mEPN shown on the left, SBGN glyphs on the right. **(A) **Shows the main symbols used for depicting biological entities and **(B) **the different ways the two schemes represent protein complexes. **(C) **Different way of showing edge meaning and **(D) **the different symbols used to depict various processes. mEPN names for these entities/activities given alongside and SBGN names, when different, in brackets beneath. For a more in depth comparison of the two notation schemes see Additional file 4.

Running concurrently with our work has been an ongoing community effort to establish rules for best practice in pathway depiction. The Systems Biology Graphical Notation http://www.sbgn.org/ project has been discussing issues and ideas around this topic and a manuscript describing the SBGN Process Diagrams Level 1 specification was recently published [43]. The mEPN scheme as described here aspires to many of the same goals as the SBGN and where possible we have tried to harmonize the mEPN scheme to the emerging SBGN specification. However, our biologist centric approach to this problem, combined with a lack of flexible pathway editing tools, the scale our diagrams and the range of biological systems we have attempted to map, have all played their part in determining the design and implementation of the mEPN scheme. As a result there are a number of important differences that exist between the mEPN as described here and the SBGN scheme for process description language as currently proposed (level 1, version 1.1). Firstly, in common with the proposed SBGN scheme, the mEPN uses glyphs of a specific shape to define the class of a component although there are some differences between the two schemes (Figure 6a). However, under the SBGN scheme the glyph representing a multimeric protein complex is comprised of each protein in a complex being depicted separately, modifications to them being overlaid on top of these and the whole thing is enclosed by a container node. We have found this a considerable overhead to implement and can interfere the clarity of what is depicted rather than enhancing it (Figure 5b). Furthermore the notation scheme is not supported by many of the general purpose network visualization tools e.g. yEd, Cytoscape, Biolayout *Express*^3D ^[44-46] in general use, requiring instead the use of dedicated pathway software. Given the relatively recent publication of the SBGN specification tools to support its deployment are largely still under development. As a result the mEPN scheme generally uses a single standard shape to depict a component even when made up of more than one entity or a series of attached entities (Figure 2d &2e). It relies on a labelling system to define the exact identity and make up of the component and its state e.g. the protein subunits that make up protein complex and their modifications (Figure 6b). Secondly, we have avoided the use of different arrowheads to depict the nature of interactions (edges). The meaning of numerous arrowheads can be challenging to remember and again they are not always supported by general pathway/network editing software packages. Instead mEPN uses inline annotation nodes to depict the meaning of edges which carry a letter symbolizing the meaning of the edge e.g. A for activation, I for inhibition, and may also use colour as an additional visual clue (Figure 6c). In principle this approach could support a wide range of edge meanings but in practice we have found many of the edge concepts supported by SBGN of no use in our mapping efforts and hence have not been included in the mEPN scheme. For instance a *consumption arc *(edge) as defined by SBGN is 'used to represent the fact that an entity affects a process, but is not affected by the process' and a *production arc *is 'used to represent the fact that an entity is produced by a process.' In the first instance, then this is the case with many enzymes acting on their substrate and in the second instance it is obvious by the fact that one thing leads on to another. In both cases we see this information as self-evident with no need for specific notation to depict it. In the case of the inclusion of specific edges to define a 'modulation' then the question is what kind of modulation is this and how would one interpret or model such a vague concept and the mEPN equivalent of the 'stimulation' edge is an activation edge. Finally, mEPN uses labelled process nodes to explicitly state the nature of interactions between components. In the proposed SBGN scheme process nodes are used, but generally not as a means to convey the nature of interactions except in the case of protein binding (association) and dissociation (Figure 6d). Whilst this approach is understandable on the basis that most process nodes would function similarly during computational modelling of such systems, not depicting the nature of the process whereby one component is transformed to another does impair visual interpretation of the diagrams. Therefore the mEPN provides a visual clue as to the nature of interaction using a one-to-three letter key to represent the nature of the process being depicted. When pathways are large and the distance between interacting species may be great, this can be an important visual aid to reading the diagrams. There are a number of other differences between the two schemes and full description of the differences between the SBGN level 1 notation and the mEPN described here can be found in Additional file 4. Whilst on these and other points the mEPN and SBGN schemes may differ, we are fully supportive of the principle of promoting the adoption of a common notation system for pathway depiction and hope that current the work will contribute to this end.

There are significant efforts already underway to garner the support and interest of the wider biological community in assembling resources, information and pathway diagrams covering a broad spectrum of biology. Indeed, the need has never been greater for these resources. However, if they do not record pathways in a standardized way, integration of the results of these efforts will continue to be a considerable issue. To this end we are fully supportive of the SGBN's effort to promote the principles of standard notation systems even if we can not fully support the proposed SBGN specification for process diagrams. We present this work and accompanying website http://www.mepn-pathway.org/ in the hope that it is as positive contribution to the debate about how best to graphically model pathway knowledge.

## Authors' contributions

TCF oversaw and contributed to the development of the mEPN scheme, has directed the development of improved computational resources to support the scheme and drafted the manuscript; SR has been instrumental in the development of many of the pathway diagrams that have driven the evolution of mEPN scheme and has contributed writing of the paper; AT has been developing the program BioLayout *Express*^3D ^to enhance its capabilities to support the visualization of pathways drawn using the mEPN scheme and their integration with data; PG oversaw the original development of the EPN scheme and supported the current development.

## Supplementary Material

Additional file 1 **mEPN Scheme Specification Document**. mEPN scheme specification document detailing each glyph and rules for their use. Click here for file

Additional file 2 **mEPN Scheme Palette**. Palette of mEPN glyphs for import into yED graph editor. Click here for file

Additional file 3 **Simple mEPN Worked Examples**. Some simple examples of mEPN notation use. Click here for file

Additional file 4 **Comparison of mEPN to SGBN**. Comparison of mEPN and SGBN schemes. Click here for file
