# Supplementary material for: The mEPN scheme: an intuitive and flexible graphical system for rendering biological pathways
Source: BMC Syst Biol. 2010 May 17;4:65. doi: 10.1186/1752-0509-4-65 (PMC2878301; doi:10.1186/1752-0509-4-65)
Supplement: Additional file 1 — mEPN Scheme Specification Document. mEPN scheme specification document detailing each glyph and rules for their use. [file 1752-0509-4-65-S1.PDF]

The modified Edinburgh Pathway Notation Scheme (mEPN) Specification Document

Description of the Notation Scheme and its Deployment

Release no 1.1
Date: July 2009

Authors:
Tom C. Freeman[1,2]
Peter Ghazal[1,3]
Sobia Raza[1,2]

Contributors:
Paul A. Lacaze[1], Kevin Robertson[1,3], Steven Watterson[1,3], Neil McDerment[1,2], Ying Chen[1], Michael Chisholm[1], George Eleftheriadis[1], Holly Gibbs[1], Stephanie Monk[1], Maire O'Sullivan[1], Arran Turnbull[1]

[1]Division of Pathway Medicine, University of Edinburgh, The Chancellor's Building, College of Medicine, 49 Little France Crescent, Edinburgh, United Kingdom. EH16 4SB.

[2]The Roslin Institute, University of Edinburgh, Roslin, Midlothian, Scotland, UK, EH25 9PS

[3]Centre for Systems Biology, University of Edinburgh, Darwin Building, King's Building Campus, Mayfield Road, Edinburgh, United Kingdom. EH9 3JU

This document describes the symbols used in the modified Edinburgh Pathway Notation (a graphical notation scheme for the depiction of biological pathways) and rules for its use. The underlying principles of the notation scheme are also explained, together with examples of its use.

Content:

1 **Introduction**

1.1 Preface
1.2 Background to Pathway Depiction

2 **Definitions and Terminology**

2.1 Pathway, Component, Interaction, Process and Event
2.2 Notation Scheme
2.3 Glyphs (Nodes)

3 **Description of mEPN Glyphs (Nodes)**

3.1 Components
3.1.1 Peptides, Proteins and Protein Complexes
3.1.2 Gene
3.1.3 DNA Sequence Feature
3.1.4 Simple Biochemical
3.1.5 Generic Entity
3.1.6 Drug
3.1.7 Ion/Simple Molecule

3.2 Process Nodes
3.2.1 Binding
3.2.2 Oligomerisation
3.2.3 Cleavage
3.2.4 Auto-cleavage
3.2.5 Dissociation
3.2.6 Catalysis
3.2.7 Auto- Catalysis
3.2.8 Translocation
3.2.9 Transcription/Translation
3.2.10 Activation
3.2.11 Inhibition
3.2.12 Phosphorylation
3.2.14 De-Phosphorylation
3.2.15 Auto-Phosphorylation
3.2.16 Phospho-Transfer
3.2.17 Ubiquitinisation
3.2.18 Sumoylation
3.2.19 Selenylation
3.2.20 Glycosylation
3.2.21 Prenylation
3.2.22 Methylation
3.2.23 Acetylation
3.2.24 Palmitoylation
3.2.25 Protonation
3.2.26 Sulphatation
3.2.27 Pegylation
3.2.28 Myristoylation
3.2.29 Oxidisation
3.2.30 Hydroxylation
3.2.31 Secretion
3.2.32 Sink (Proteasomal Degradation)

3.3. Other Nodes
3.3.1 Energy / Molecular Transfer
3.3.2 Conditional Switch
3.3.3 Pathway Module
3.3.4 Pathway Output

3.4 Boolean Logic Operators
3.4.1 & Operator
3.4.2 OR Operator

4 Edge Use and Depiction of Interactions between Components
4.1 Description of Edges
4.1.1 Interaction
4.1.2 Physical Link
4.1.3 Interaction - details unknown
4.1.4 Pathway Input
4.1.5 Pathway Output
4.1.6 Activates
4.1.7 Inhibits
4.1.8 Catalysis

5 Cellular compartment
5.1 Depiction of Cellular Compartments

6 Use of Colour
6.1.1 Colouring Components by Type
6.1.2 Colouring Components by Location
6.1.3 Colouring Components to Reflect Biological Data

7 Annotation of Pathway Networks

8 Layout Rules for Modified Edinburgh Pathway Notation

9 mEPN 3D Scheme and Visualisation of Pathway Information in 3D Environment

10 References

11 Appendix

Appendix Figure 1
Appendix Figure 2
Appendix Figure 3

1 Introduction

1.1. Preface

The modified Edinburgh Pathway Notation (mEPN) is founded on a notation system originally proposed by Moodie *et al.* 2006[1] and first published in a form similar to that described here by Raza et al. 2008[2]. Its recent evolution and refinement has been primarily driven by the author's attempts to produce process diagrams[3] for a diverse range of biological pathways, particularly with respect to immune signalling in mammals. These efforts have also been influenced by the work of the Systems Biology Graphical Notation (SBGN) community[4] and others in the field. The current mEPN scheme, the rules for its deployment and creation of a number of large pathway diagrams has been a collaborative effort between members of the Division of Pathway Medicine, the Roslin Institute and the Edinburgh Centre for Systems Biology, University of Edinburgh.

1.2. Background to Pathway Depiction

Pathway diagrams act as a visual representation of known networks of interaction between cellular components. Modelling of pathways is fundamental to our understanding the workings of biological systems however the task of assimilating the large amounts of available data and representing this information in an intuitive manner remains a challenge. Accordingly there has been increasing interest in the biology community to develop approaches for representing biological pathways. The Molecular Interaction Map (MIM)[5,6] and Process Description Notation schemes[3] were proposed by Kurt Kohn and Hiroaki Kitano, respectively, and their ideas laid the foundations for much of the work on pathway notation that has followed. The modified Edinburgh Pathway Notation scheme is based on the principles laid down for depicting process diagrams:

1. Allow the detailed representation of diverse biological entities, interactions and pathway concepts
2. Provide a system for presenting pathway knowledge in a semantically and visually unambiguous manner
3. Have network semantics that are sufficiently well defined that software tools can convert graphical models into formal models, suitable for analysis and simulation
4. Be as simple as possible to read and use
5. Understandable to a biologist

The current mEPN scheme is the based on the experience of over four years of pathway construction, notation testing and discussions. In this document we define the mEPN scheme and describe its use for depicting biological pathways. The objectives of the EPN as originally proposed remain preserved as do many of its original concepts of the EPN scheme[1]. However substantial modifications have been made to the notation system from the introduction of new symbols to changes in the aesthetics of the scheme and pathway syntax.

2 Definitions and Terminology

Below we define the commonly used terminology within the document.

2.1. Pathway:

A directional network of molecular **interactions** between **components** of a biological system that act together to regulate a cellular **event** or **process**.

Where:

- **Components** are any entity involved in a pathway be it a protein, protein complex, nucleic acid (DNA, RNA), molecule, cellular structures etc.

- **Interactions** are generally the relationships between one component and another where one component influences the activity of another through its binding to, inhibition of, catalytic conversion of etc.

- An **Event** can be defined as a change in a biological system triggered by an alteration in the environmental conditions or presence of biologically active factors: infection, molecular signalling, genotype, temperature, oxygen/water/salt balance, nutrient change etc.

- A **Process** is a defined event occurring that is related to either metabolism, development, disease, immunology etc. They can be a specific reactions or general processes.

2.2. Notation Scheme

A collection of predefined symbols (shapes, lines, figures) that represent the constituent parts of a graphical system for depicting the components of a biological pathway, the interactions between them and the cellular compartments in which they occur. A scheme also includes rules for the use of these symbols.

2.3. Glyphs (Nodes):

A glyph is stylised graphical symbol that imparts information nonverbally. They are used here to portray different classes of biological entities e.g. protein, gene, pathogen etc. and the nature of the relationship between them. In network terminology all glyphs are a node of a specific type and the connectivity between them defined by edges (lines/arcs).

3 Description of mEPN Glyphs (Nodes)

3.1 Components

3.1.1 Peptides, Proteins and Protein Complexes

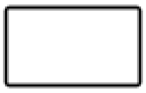

Glyph: rounded rectangle

Any peptide, protein or protein complex.

Annotation
Protein names: standard gene names (e.g. HGNC, MGD) used to describe protein depicted. Where other names (alias') are in common use these name(s) may be shown as an addition to the label on the glyph representing the protein (only where it first appears on the pathway as an individual component) after the official gene symbol in rounded ( ) brackets.

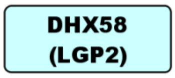

Protein complex names are given as a concatenation of the proteins belonging to the complex separated by a colon. If the complex is commonly referred to by a generic name this may be shown below the constituent parts. There are no strict rules as the order in which the names are given and are often shown in the order in which the proteins join the complex, in the position they are likely hold relative to other members of the complex (where known) or position relative to cellular compartments e.g. with receptor proteins in a membrane bound protein complex protruding into the extra-cellular space. Note, caution should be taken to avoid representing the same complex twice with the order of the constituent proteins in a different arrangement.

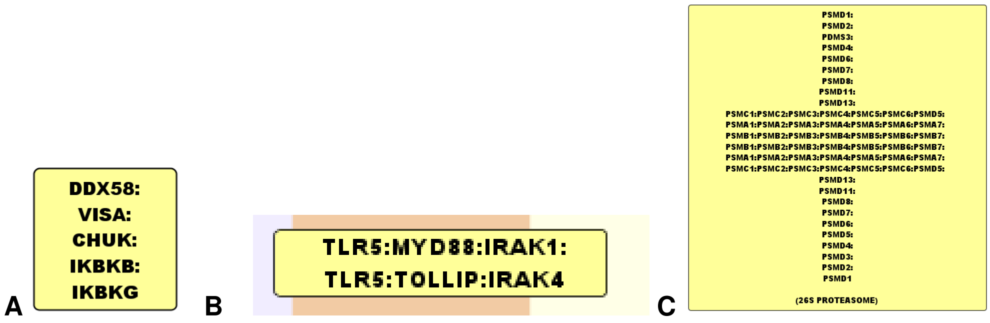

Examples of the Depiction of Protein Complexes. **A.** Simple depiction of complex where constituent subunits are given as a list separated by a colon. **B.** Example of a plasma membrane receptor complex where subunits are arranged so as to allow the complex to span

the membrane having elements projecting into the extracellular space as well as the cytoplasm. **C.** The 26S proteasome where we have attempted to show something of its complex structure by arranging the subunits in layers (representing the barrel of the proteasome) and the regulatory cap structures.

Multimeric protein complexes. Where a specific protein is present multiple times within a complex, this may be represented by placing the number of times a protein is present within the complex in angle brackets < > e.g. the apoptosome. If the number of proteins in the complex is unknown this may be represented by <n>.

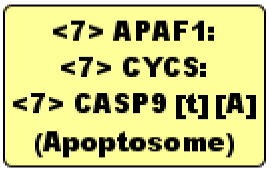

Example of multimeric protein complex, the active apoptosome consists of 7 APAF1 proteins, 7 CYCS proteins, and 7 truncated CASP9 proteins.

Protein state: The particular ‘state’ of an individual protein or a protein within a complex may be altered as a consequence of a particular process. This change in the component’s state is marked using square [ ] brackets following the component’s name; each modification being placed in separate brackets. This notation may be used to describe the whole range of protein modifications from phosphorylation [P], truncation [t], ubquitinisation [Ub] etc. (see notation key Appendix Figure 1 for full range). Where details of the site of modification are known this may be represented e.g. [P-L232] = phosphorylation at leucine 232. Alternatively the details of a particular modification may be placed as a *note* on the node. Where multiple sites are modified this may be shown using multiple brackets, each modification (state) being shown in separate brackets.

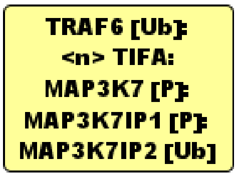

Example of a protein complex with multiple modifications. TRAF6 and MAP3K7IP2 are both ubiquitinated. MAP3K7 and MAP3K7IP1 are both phosphorylated.

3.1.2 Gene

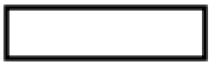

Glyph: rectangle

Used to denote transcribed genomic DNA locus encoding a protein.

Annotation: Named according to standard gene names (e.g. HGNC, MGD).

3.1.3 DNA Sequence Feature

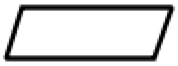

Glyph: parallelogram

A specific DNA sequence known to play a specific functional role e.g. promoter sequence. This may be shown on its own or associated with a gene or other genomic feature.

Annotation: Named according to common name of site. Specific details e.g. sequence may be added as node *notes*.

3.1.4 Simple Biochemical

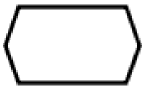

Glyph: hexagon

Used to represent a defined simple biochemical molecule e.g. sugar, amino acid, nucleic acid, metabolite.

Annotation: There appears to be no universally recognised nomenclature systems for many of these classes of molecules, we have therefore generally names commonly used amongst biologists.

3.1.5 Generic Entity

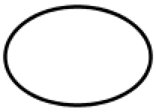

Glyph: Ellipse (oval)

Depicts a generic class of components e.g. pathogen, bacteria, or a class of molecular species e.g. DNA, LPS.

Annotation: Name used commonly amongst biologists.

3.1.6 Drug

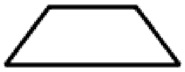

Glyph: trapezoid

Any small molecule or biologic known to affect a biological system. These may be licensed as a drug or used for experimental manipulation of biological components e.g. enzyme inhibitor, siRNA etc.

Annotation: Name used commonly amongst biologists and pharmacologists.

3.1.7 Ion/Simple Molecule

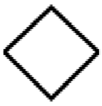

Glyph: Diamond

Used to represent an ion e.g. Ca^{2+}, Na^{+}, Cl^{-}, or simple inorganic molecule e.g. H_2O, NO, O_2, CO_2. Due to the ubiquitous nature of such entities they may be represented more than once in any given compartment.

Annotation: Name standard chemical symbol.

Note on Component Colour
A node representing a component may be coloured to impart information on components type, location or state e.g. to visually differentiate between a protein and a complex, to denote cellular location or denote a component's expression level. [See section **Use of Colour**]

3.2. Process Nodes

A Process Node in the context of this notation system can be defined as a node that infers an action, transformation, transition or process. They impart information on the type of process that is associated with transformation of a component from one state to another or movement in cellular location. They may act as junctions between components and as such may have multiple inputs or outputs to components.

All process nodes are represented by circular glyph and the process they represent is defined by a one-to-three letter code. Colour has been used as a visual clue to group processes into 'type' but is not necessary for inferring meaning.

Definition of Individual Process Nodes:

3.2.1 Binding

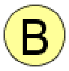

The physical association (binding) of two or more components through covalent or non-covalent interactions.

3.2.2 Oligomerisation

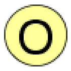

The physical association (binding) of two or more identical polypeptides resulting in an oligomeric-complex e.g. homodimer, homotrimer etc.

3.2.3 Cleavage

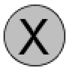

The splitting of a polypeptide into smaller fragments, usually through the action of another protein (enzyme) or protein complex.

3.2.4 Auto-cleavage

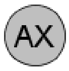

The splitting of a polypeptide into smaller fragments by itself or by the action of protein within the same complex as the protein undergoing cleavage.

3.2.5 Dissociation

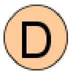

The separation of a protein or group of proteins from a protein complex.

3.2.6 Catalysis

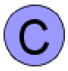

The catalytic conversion of a component from one state to another by an enzyme. Used generically to depict the transformation of a biomolecule from one form to another. Certain types of common catalytic conversions have their own process node e.g. phosphorylation, ubiquitinisation etc.

3.2.7 Auto- Catalysis

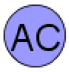

The catalytic conversion of a component from one state to another that is facilitated by the same component or subunit thereof.

3.2.8 Translocation

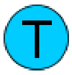

The movement of a component from one sub-cellular compartment to another. Translocation nodes are drawn at the intersection between compartments and the lines entering and leaving the node coloured blue to emphasise visually the transition.

3.2.9 Transcription/Translation

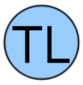

Used to link a node representing a gene with corresponding protein node. Infers gene transcription and the recruitment and assembly of amino acids to form a peptide chain based on the mRNA sequence.

3.2.10 Activation

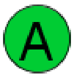

The conversion of a component from a latent/inactive state to a functionally state. The use of this node usually infers that the details of this process are not known or have not been captured.

3.2.11 Inhibition

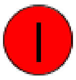

The inhibition or inactivation of a component.

3.2.12 Phosphorylation

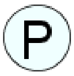

The addition of a phosphate group to a protein or protein complex.

3.2.13 De-Phosphorylation

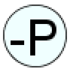

The removal of a phosphate group from a protein or protein complex.

3.2.14 Auto-Phosphorylation

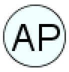

The addition of a phosphate group to a protein or protein complex which is catalysed by the same protein or protein complex.

3.2.15 Phospho-Transfer

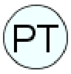

Movement of phosphate-groups during a signalling reaction. A mechanism of molecular communication between a sensor component and a phospho-accepting component principally based on histidine-to-aspartate (His-Asp) phosphor-transfer.

3.2.16 Ubiquitinisation

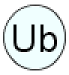

The attachment of one or more ubiquitin monomers to a protein or protein complex. Mono-ubiquitinisation is often used to regulate the activity of proteins/protein complexes, poly-ubiquitinisation often leads to the proteasomal degradation of the tagged protein.

3.2.17 Sumoylation

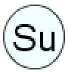

The attachment of SUMO (Small Ubiquitin-like Modifier) protein to proteins or complexes (usually to modify their function or activity).

3.2.18 Selenylation

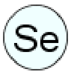

The attachment of a selenium element to a component.

3.2.19 Glycosylation

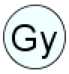

The addition of glycosyl groups to a protein or complex.

3.2.20 Prenylation

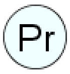

The addition of a prenyl group (farnesyl (15-carbon) or geranylgeranyl (20-carbon) isoprenoids) to cysteine residues or the c-terminus of proteins or complexes (causing lipid modification).

3.2.21 Methylation

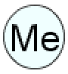

The attachment of a methyl group to a component.

3.2.22 Acetylation

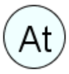

The attachment of an acetyl group to a component.

3.2.23 Palmitoylation

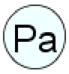

The attachment of fatty acids (such as palmitic acid) to cysteine residues of proteins or complexes.

3.2.24 Protonation

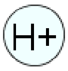

The addition of a proton (H^+) to a component.

3.2.25 Sulphatation

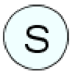

The addition of a sulphate group to a component.

3.2.26 Pegylation

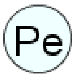

The covalent attachment of polyethylene glycol polymer chains to a component.

3.2.27 Myristoylation

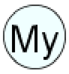

The attachment of a myristoly group to the N-terminal of a protein usually during protein translation

3.2.28 Oxidisation

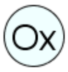

The addition of an oxygen molecule to a component.

3.2.29 Hydroxylation

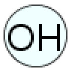

The addition of a hydroxyl group (OH) to a component usually at a proline residue in proteins/ protein complexes.

3.2.30 Secretion

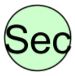

The secretion of a protein or a biochemical out of the cell and into the extracellular space. Replaces use of translocation node when a component moves from the cytoplasm to the extracellular space.

3.2.31 Sink (Proteasomal Degradation)

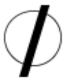

Removal of a component from the system/pathway, usually by proteasomal degradation. In principle this symbol can also be used to denote a component joining a system by formation from constituent parts although this has never been used by us to represent this.

3.3 Other Nodes

3.3.1 Energy/ Molecular Transfer

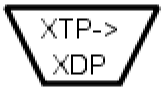

Glyph: Trapezoid

Simple co-reaction associated with the process (e.g. ATP→ADP, GTP→GDP, NADPH→NADP$^+$) needed to drive certain reactions.

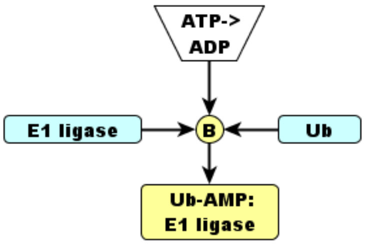

e.g. The binding process of E1 Ligase to Ub requires ATP → ADP.

3.3.2 Conditional Gate

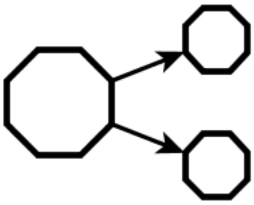

Glyph: Combination (octagon connected to two or more smaller octagons)

A conditional gate is used where there are potentially multiple fates of a component and the output is dependant on other factors such as the components concentration, time or is associated with a cellular state.

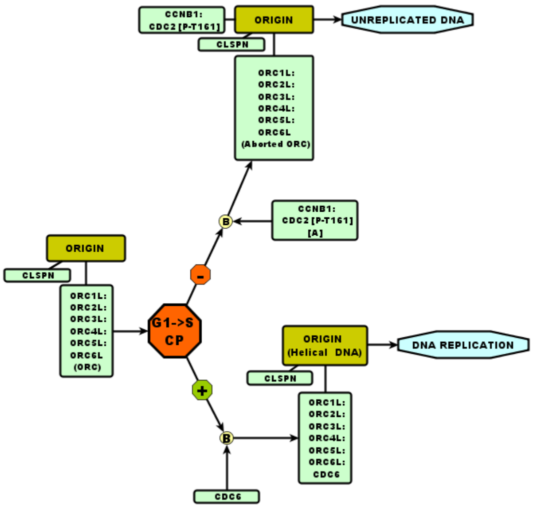

Example of the use of a conditional gate. Shown here is one of the checkpoints for the G1 to S phase transition of the cell cycle. Following the formation of the ORC/CLSPN complex at an origin of replication, two outcomes are possible. If conditions are favourable CDC6 will bind to the ORC complex and this is an initiating step in DNA synthesis. However where conditions are not favourable a complex of CCNB1:CDC2 binds and DNA synthesis is aborted. In this instance the factors determining DNA synthesis progression or not, are not clear.

3.3.3 Pathway Module

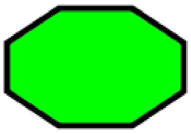

Glyph: Compressed octagon

Pathway modules define complicated processes or events that are not otherwise fully described. Examples include signalling cascades, endocytosis, compartment fusion etc.

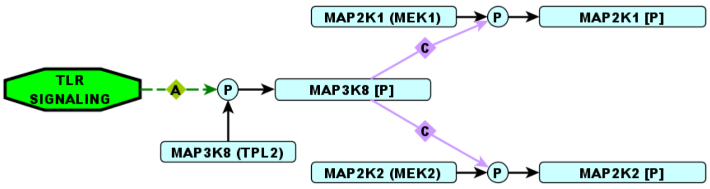

A Pathway Module depicting TLR signalling activation of the ERK MAPK pathway.

3.3.4 Pathway Output

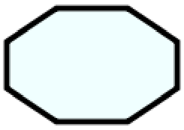

Glyph: Compressed octagon

A pathway output details the cumulative output of series of interactions or function of an individual component at the end of a pathway. Pathway outputs are shown in order to describe the significance of those interactions in the context of a biological process or with respect to the cell. The input lines leading into a pathway output node have been coloured light blue to emphasise the end of the pathway description.

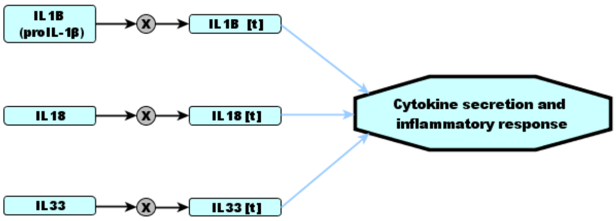

Example of the use of a pathway output mode. Activation of the inflammasomes leads their catalytic cleavage of one or a number of interleukins thereby activating them. In this instance the specific differences in the actions of these three cytokines has not been stated.

3.4 Boolean Logic Operators

Boolean logic operators define the dependencies between components of a system. They are used to define the relationships between multiple inputs into a process.

3.4.1 & Operator

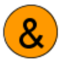

An AND operator is used when two or more components are required to bring about a process i.e. an event is dependent on more than one factor being present.

3.4.2 OR Operator

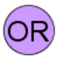

An OR operator is used when one component or another may cause the same change in another component. This operator is used to form an intersection between the interaction edges emanating from the reacting components.

4 Depiction of Interactions between Components and the Use of Edges

Edges denote that an interaction occurs between components/process in a pathway and the directionality of that interaction. The nature of an interaction is inferred through the use of process nodes, Boolean logic operators and edge annotation nodes. Interaction edges may be coloured for visual emphasis but as with nodes, the definition of meaning is not reliant on colour. A number of edges contain an in-line annotation to indicate the type of interaction as is sometimes depicted by the use of different arrow heads. An edge annotation is generally characterised as having only one input and one output and functions to describe the type of activity implied by the line e.g. translocation, activation, inhibition, catalysis. However in certain instances they can be used as distribution nodes e.g. where one component activates many others such as with transcriptional activation of a number of genes by a transcription factor it can reduce the number of edges emanating from the TF. Use of differing arrow-heads has been avoided altogether for several reasons; firstly, there is a limit to the number of differing types of arrowheads which potentially falls below the possible number biological concepts one may need to depict. Secondly, differentiating between a number of different arrow-heads is sometimes difficult when viewed at a distance. Thirdly, few arrow-heads are symbolic or indicative of the action they are designed to describe requiring them to be committed to memory. Finally, multiple arrowhead types are not always supported by different network-editing/visualisation software.

4.1. Description of Edges

4.1.1 Interaction

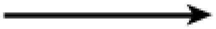

Defines a directional link (input or output) between nodes of a pathway be they components, process nodes, logic operators.

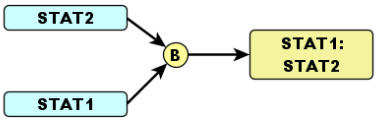

Input edges leading from STAT1 and STAT2 proteins feed into the process node denoting their binding. An output edge from the binding node links to the output of this process the complex STAT1:STAT2.

4.1.2 Physical Link

An undirected edge denotes a physical connection (bond) between two or more components where separate depiction of modules belonging to the same component is required.

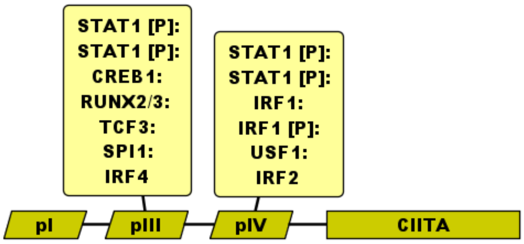

Promoter of CIITA with bound transcription factor complexes. Here we have used the physical link edge to denote the attachment of the known promoter regions/sequences of CIITA to the genes coding sequence, as well as the bound activating complexes.

4.1.3 Interaction - Details Unknown

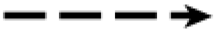

A dashed interaction edge can be substituted in place of any of the above ‘interaction edges’ where the precise details and nature of the interactions are unclear.

4.1.4 Pathway Input

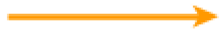

'Pathway Input' helps visually to define the start of a pathway i.e. the first in a series of events.

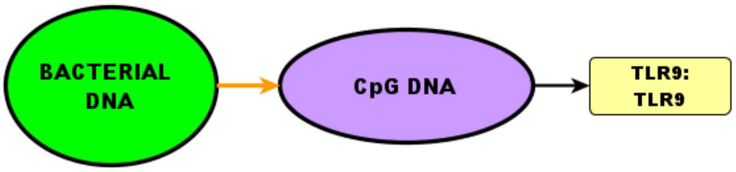

Bacterial DNA marks the start of the TLR9 signalling pathway

4.1.5 Pathway Output

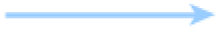

'Pathway Output' edge defines a conclusion of a pathway and is always used in conjunction with a 'Pathway Output' node which describes the event and/or conclusion to a pathway.

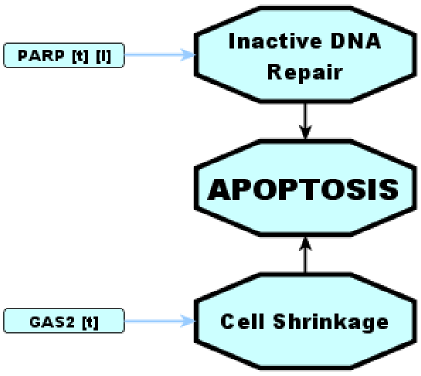

Truncated PARP and GAS2 result in the outputs of inactive DNA repair and cell shrinkage which leads to apoptosis.

Note: The following three edge types employ the use of an inline annotation node that provides a visual definition of the edge type. This approach allows for the support of a potentially large repertoire of edge meanings. In these cases the edge annotation node only ever possesses one input possessing no arrowhead and the direction of the edge being indicated only when it reaches its target. As a visual aid the edge(s) are coloured the same as the node.

4.1.6 Activates

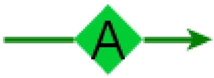

'Activates' edges are used to infer that that one component activates another or functions to activate a process. It does not however infer anything of the mode of action of this activation either because it is not known or has not been captured.

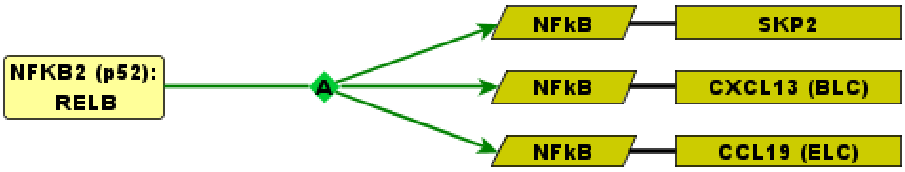

Activation of 3 genes by NFKB2 (p52):RELB complex through NFkB binding site.

4.1.7 Inhibits

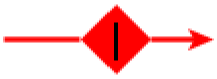

'Inhibits' edges are used to infer that that one component inhibits another or functions to inhibits a process. Similar to activation edges they do not infer anything of the mode of action of this inhibition either because it is not known or has not been captured.

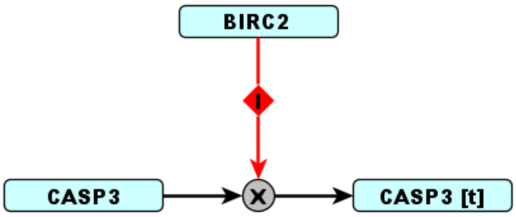

BIRC2 inhibits the process of CASP3 activation by preventing its cleavage into the truncated form of the protein.

4.1.8 Catalyses

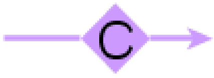

A 'Catalyses' edge connects a component to a process node where the component is responsible for catalysing the process depicted.

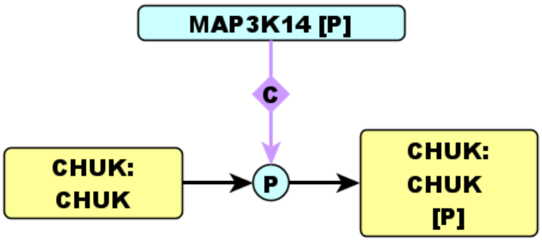

Phosphorylated MAP3K14 (NIK) catalyses the phosphorylation of the CHUK:CHUK homodimer complex.

Note on Process Nodes, Logic Operators and Edge Annotation Nodes
Nodes representing any of the above depict concepts and meanings concerning the interaction between components. Therefore they do not represent physical entities and as such do not strictly exist in any location even if depicted as belonging to one or another compartment. We have developed a colour scheme to help visually distinguish these nodes but their meaning is entirely supported by the use of the 1-3 letter code.

5 Cellular compartment

A cellular compartment can be a region of the cell, an organelle or cellular structure, dedicated to particular processes and/or hosting certain sub-sets of components e.g. genes are found only in the nuclear compartments.

5.1 Depiction of Cellular Compartments

Sub-cellular compartments are defined by a labelled pathway background and arranged with spatial reference to a cell. Compartments are coloured differently for emphasis and to ease awareness the location of components. A proposed colour scheme for compartments is shown in Appendix Figure 1. Similar or related compartments share the same fill colour but have different coloured perimeters to define internal boundaries within a compartment e.g. membrane vs. lumen or to define the origin of compartments e.g. different classes of vesicles derived from the endoplasmic reticulum or plasma membrane.

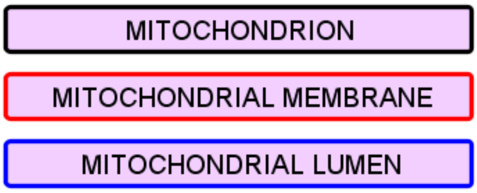

Colour scheme used for related sub-cellular compartments. The core colour for related compartments is the same however the perimeters of the compartments have different colours.

6 Use of Colour

The mEPN scheme has been designed to function in the absence of colour and no aspect of it is dependant on colour for its full understanding, hence avoiding issues variable colour recognition capabilities between individuals and issues with a poor reproduction of figures. However colour is a powerful visual tool and has been used in the deployment of the mEPN for emphasis. A proposed colour scheme is described below and in Appendix Figure 1 but is open for adaptation to suit the end users needs or aesthetic tastes. Nodes may be coloured to differentiate between different node types e.g. between a protein, complex or gene, to denote their cellular location or expression/activity level.

6.1. Colouring Components by Type

Colouring nodes by type can ease the differentiation of different components. Below is the colour scheme used whereby components are coloured by type. The colour scheme in use for Process nodes, Boolean operators and edges can be seen in Appendix Figure 1 and within this document at the corresponding sections.

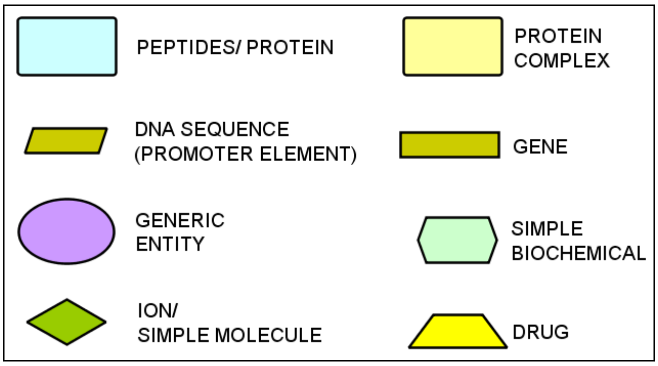

6.1.2 Colouring Components by Location

Components can also be coloured by their sub-cellular location using the same colouring scheme that applies for colouring cellular compartments. If one chooses an alternative layout to view a pathway where the original spatial arrangement of the components is lost then it will still be possible to identify where the components are interacting by their colour. Hence colouring nodes by their location provides the flexibility of arranging the pathway using alternative layouts but without compromising loss of information about the sub-cellular location of components.

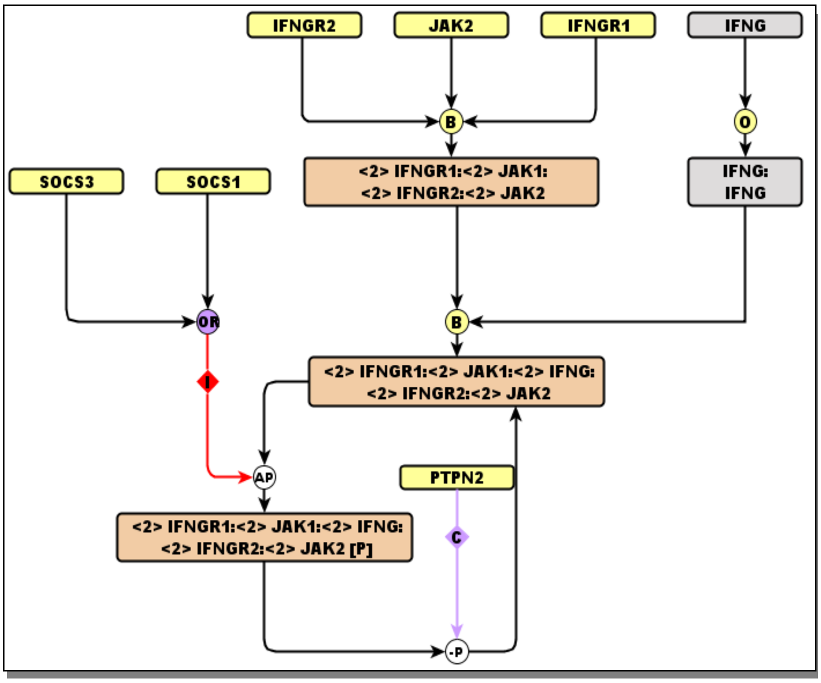

A section of the interferon pathway laid out using an automated-layout algorithm. The sub-cellular location of components is still identifiable by their colour (yellow is cytoplasm, tan is cell membrane, and grey is extra-cellular).

6.1.3 Colouring Components to Reflect Biological Data

The end-users of the pathway can define a colour scheme of choice to represent the activity of the pathway components within a data set. In the example below nodes are coloured orange if they are expressed. The spectrum or intensity of colours may be used to reflect the absolute level of expression or activity.

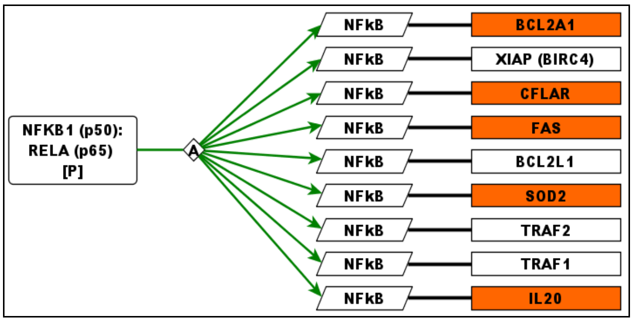

7 Annotation of Pathway Networks

Additional notes and hyperlinks links to external databases are useful in conveying additional information on pathway biology. Graphml files support this activity. In later version of our pathway diagrams PubMed identifiers are provided for each interaction depicted within the pathway diagram, as are URL-links to Entrez gene for each protein or gene component in the pathway. Furthermore descriptions obtained from either Entrez gene or RefSeq or OMIM are included for individual components (proteins/genes/compexes). Textual descriptions are included for complex interactions or to provide additional information of any aspect of the interaction and may be added by the pathway curator to supplement what is shown graphically. Additional notes from pathway curator, PubMed IDs are stored on appropriate edges or nodes and are visible in the properties-description tab for nodes or edges, or appear when hovering over an node or edge. URL-links are stored under the properties-URL tab.

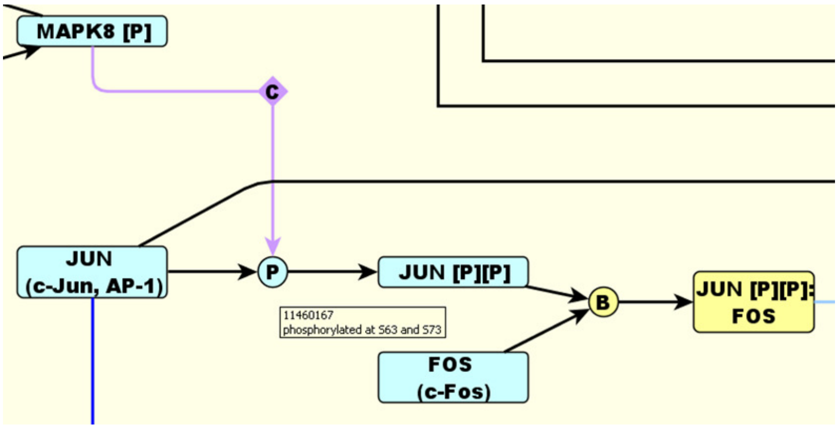

Notes on the phosphorylation of the JUN protein viewed by mouseover of process node. Included here is the PubMed identification number and the exact phosphorylation sites of JUN.

8 Layout Rules for Modified Edinburgh Pathway Notation

1. mEPN pathways are drawn as networks. Nodes either represent the components of a biological system, the nature of specific events (processes) between components or transition from one state to another. Edges connect these entities and concepts into a network. We have used the freely available yEd editor package (yFiles, Tubingen, http://www.yworks.com/) for pathway construction but in principle other network editor programs could be used.

2. Pathway components are represented by nodes (glyphs) of a specific shape. The shape of the node is determined by the type of entity, process or concept being represented e.g. round rectangle for proteins and protein complexes, flattened ellipse for gene etc, circles for processes/Boolean operators, diamonds for edge annotations (see Appendix, figure 1). The identity of components is placed inside the node. Standard nomenclatures (e.g. HGNC for human, MGD for mouse) must be used for all protein/gene names to avoid ambiguity over the identity of what is being represented. Nomenclatures from different species should not be mixed. If a protein or complex is commonly referred to by another name (alias) then the alternative name may be placed in brackets by the side or underneath of the standard name e.g. NFKB1 (p50). Protein complexes are described by the concatenation of names of the proteins that make it up. These may be supplemented by complex names in current use.

3. Component layout is performed manually and components are placed in their site of cellular activity, represented as predetermined areas (compartments) on the canvas.

4. A component may only be shown once in any given cellular compartment (in a given state).

5. A component may however alter from one state to another e.g. inactive to active, unbound to bound, in which case both forms are represented as separate entities. To indicate a different state this may be included under the name in square brackets e.g. [A] – active, [P] – phosphorylated.

    - The transformation of a component from one state/form to another or from one localisation to another is shown by use of arrows (edges), Boolean operators and process nodes.

    - Process nodes add annotation about the nature of protein interactions, state or localisation changes and are depicted as small round circles with lettering to indicate the type of process. Process nodes are used to specify the type of interaction that takes place between one component and another e.g. P – phosphorylation, B – binds, X – cleavage.

- Boolean operators are used to depict interactions logically and define dependencies between interacting components.

6. Nodes (components, processes, operators) and edges (interactions) are drawn in such a way as to make the diagram compact with a minimum about of crossing over, changes in direction of edges and length. Edges should be easy to follow. However when diagrams exceed a certain size it may be necessary to sp

7. Colour may be added to the diagrams to assist in their interpretation. Components are generally coloured according to their type e.g. protein, complex, gene, or sub-cellular localisation. Gates and edges may also be coloured to improve the visual impact of the diagram. A proposed colour scheme is shown in the notation key (reference Figure 1), however it must be stated that the exact choice of colours is down to individual taste and colour recognition capabilities.

8. Evidence of an interaction between one and component and another is stored in an interaction table. Evidence to support an interaction is derived from the primary literature (and reviews). This must include the interacting partners, the direction of the interaction is infer by order HGNC1 -> HGNC2, the type of interaction (phosphorylation, cleavage), method, PubMed ID, site of specific change of state [P-Ser123]. More than one paper may be used to support the same interaction (two or more is preferable). No interaction may be included within the pathway without published evidence. An example of a pathway interaction table is shown in reference Figure 2.

9. Hierarchical relationships between components should be shown in the layout of interactions. In order to do this an orientation of pathway flow is chosen (e.g. left to right or top to bottom) and should be maintained throughout the diagram where possible, i.e. the input of an interaction should precede the output of that reaction when following the direction the pathway flow has been set. Ideally the direction of the edges should follow the flow of the pathway information, although it is appreciated this becomes more difficult in larger diagrams.

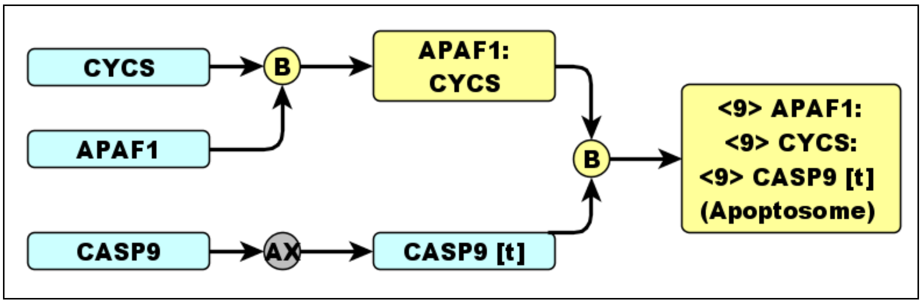

Ideal layout of interactions (when flow is set from left to right). The interactions depict the formation of the Apoptosome complex. Outputs of each process fall to the right of the process node and inputs to the left. Following the flow of information and identifying the main output or product (Apoptosome) is relatively straightforward.

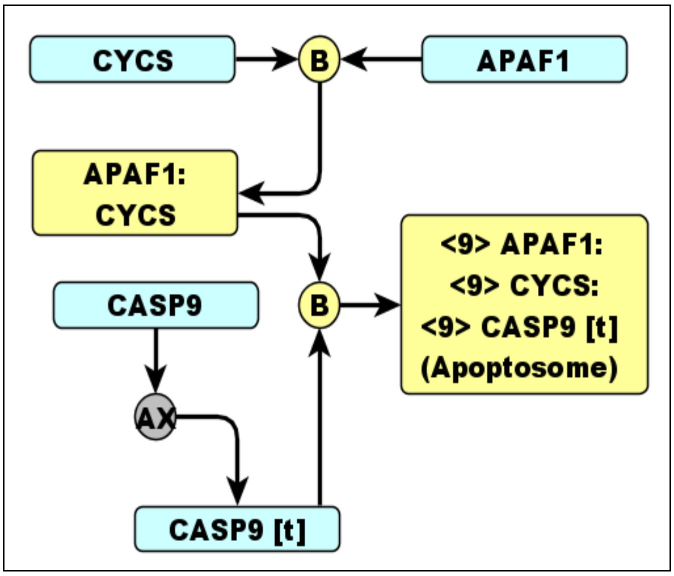

Poor layout of interactions (when flow is set from left to right). The interactions depict the formation of the Apoptosome complex. The flow of information is running from both left to right and from top to bottom. It is relatively difficult to identify where the pathway begins and what is the output from the series of interactions.

9 mEPN 3D Scheme and Visualisation of Pathway Information in 3D Environment

Layout of pathways in 3D space as network graphs begins to address the issue of scalability associated with the large pathway diagrams and offers new ways to visualise and interact with pathway diagrams. A 3D translation of mEPN scheme is shown in Appendix Figure 3. The scheme is devised to reflect the colours and where possible glyphs used in the 2D mEPN process diagrams. The notation scheme is currently supported by the network visualisation and analysis tool BioLayout *Express*^{3D} [7,8] (http://www.biolayout.org/) which currently supports the input of pathways as .graphml files. Below are a number of screenshots of our current macrophage activation pathway drawn using the standard (2D) notation scheme and imported into BioLayout *Express*^{3D}.

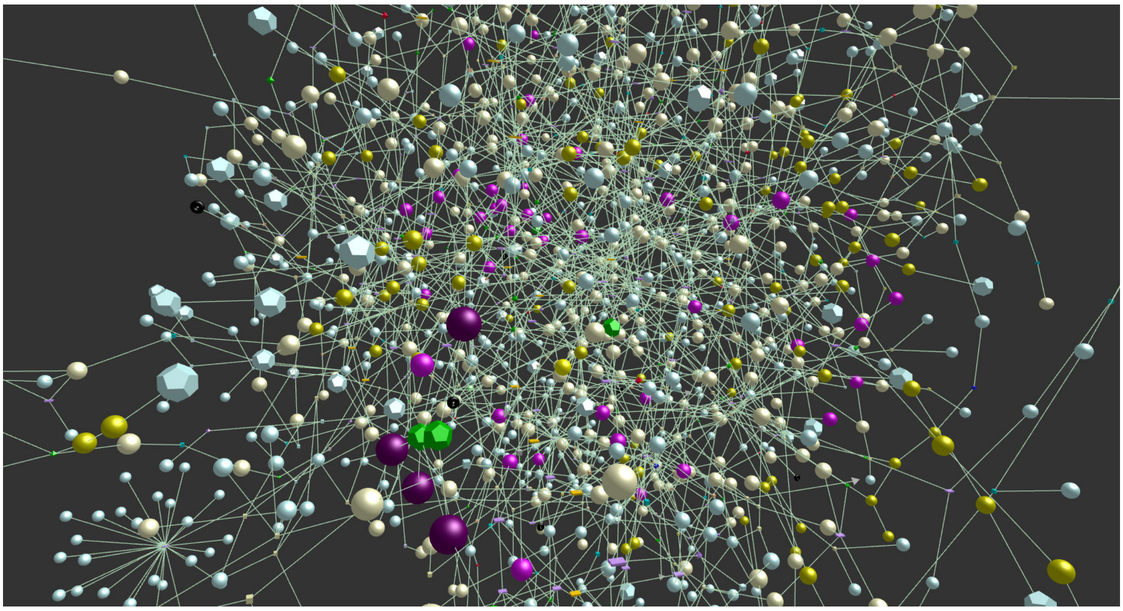

Organic (modified Fruchterman-Rheingold) layout of macrophage pathway diagram in 3D environment using mEPN 3D scheme where node shape and colour is according to node type. Pathway displayed using BioLayout *Express*^{3D} (http://www.biolayout.org/).

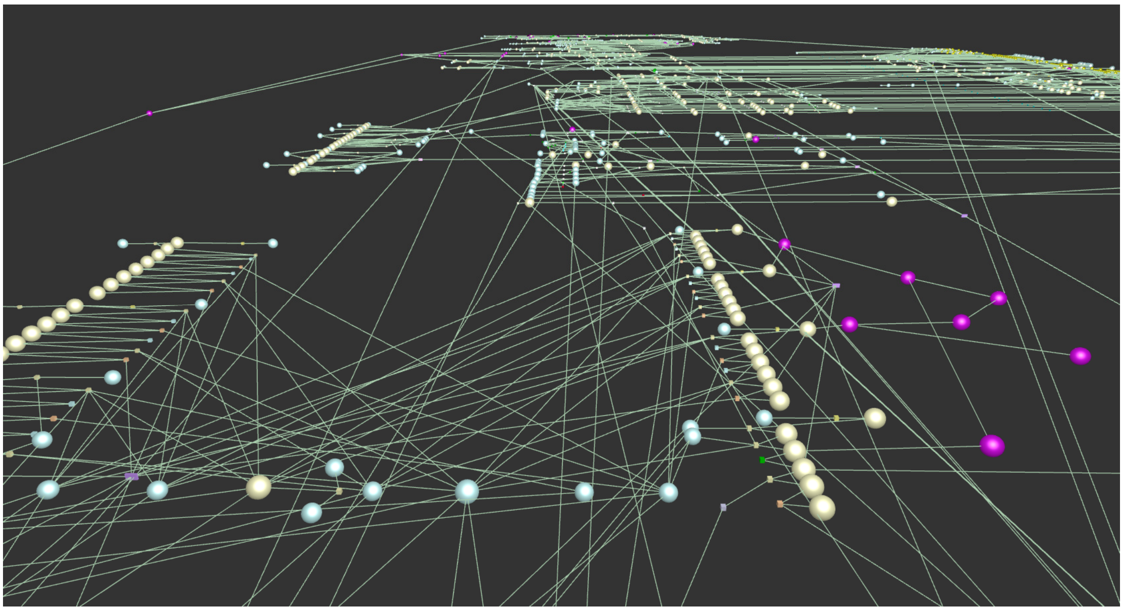

Display of macrophage pathway diagram in 3D environment using mEPN 3D scheme where node shape and colour is according to type e.g. light blue sphere – protein; off-white sphere – protein complex; purple sphere – generic-entity etc. (see Appendix 3 for full description of mEPN 3D scheme). Pathway displayed using manually curated 2D node co-ordinates taken from .graphml file of pathway.

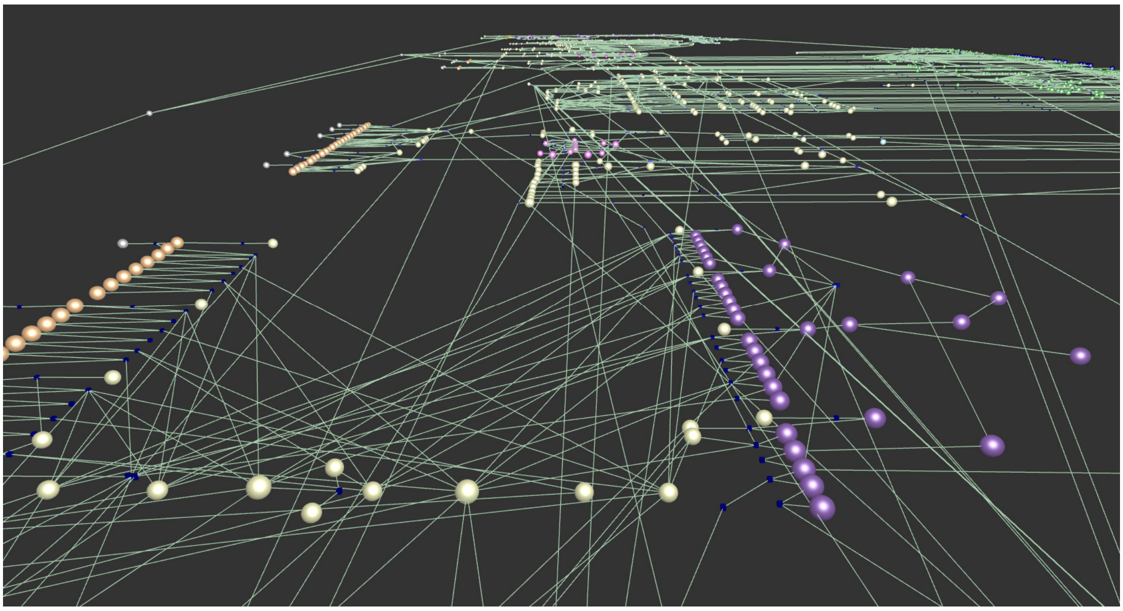

Display of macrophage pathway diagram using mEPN 3D scheme for node shape, node colour according to sub-cellular compartment e.g. cytoplasm – off-white; purple – endosome; brown – plasma membrane; green – nucleus. Process nodes, Boolean logic operators and edge annotation nodes are shown as having no location and coloured dark blue.

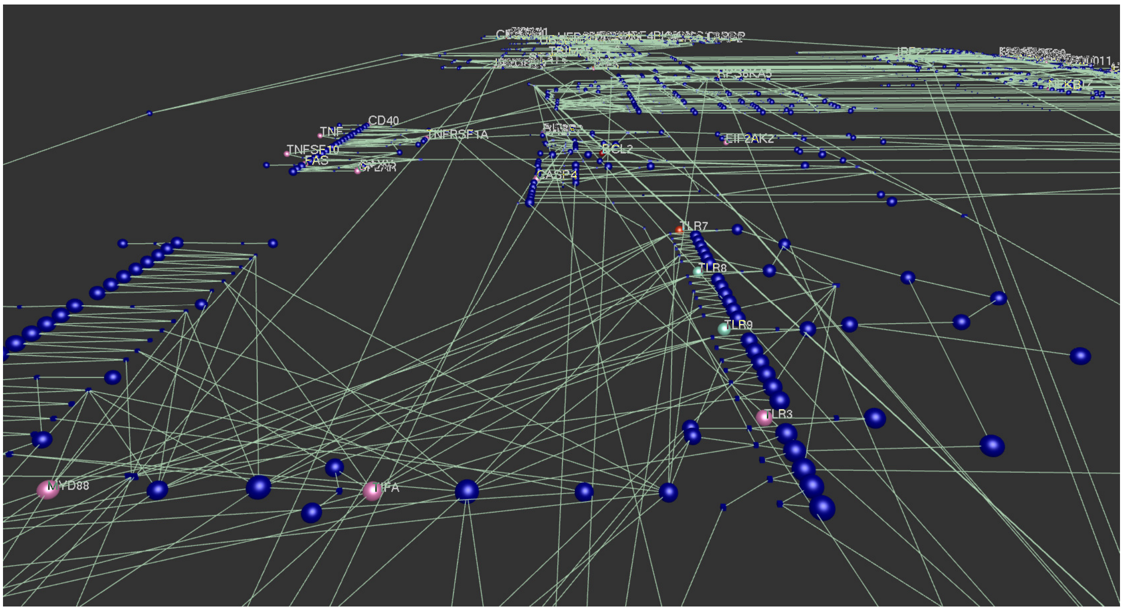

Display of macrophage pathway diagram using mEPN 3D scheme for node shape. Gene and protein nodes are coloured according to network cluster ID, as determined by transcriptional profiling of mouse macrophages after stimulation with interferon-β. All other nodes coloured dark blue.

10. References

1. Moodie S.L., S.A., Goryanin I., Ghazal P. A Graphical Notation to Describe the Logical Interactions of Biological Pathways. *Journal of Integrative Bioinformatics* **3**, 11 (2006).
2. Raza, S., Robertson, K.A., Lacaze, P.A., Page, D., Enright, A.J., Ghazal, P. and Freeman, T.C. A logic-based diagram of signalling pathways central to macrophage activation. *BMC Systems Biology*, **2**, 36 (2008).
3. Kitano, H., Funahashi, A., Matsuoka, Y. & Oda, K. Using process diagrams for the graphical representation of biological networks. *Nat Biotechnol* **23**, 961-966 (2005).
4. Le Novère N, Hucka M, Mi H, Moodie S, Shreiber F, Sorokin A, Demir E, Wegner K, Aladjem MI, Wimalaratne SM, Bergman FT, Gauges R, Ghazal P, Kawaji H, Li L, Matsuoka Y, Villéger A, Boyd SE, Calzone L, Courtot M, Dogrusoz U, Freeman TC, Funahashi A, Ghosh S, Jouraku A, Kim S, Kolpakov F, Luna A, Sahle S, Watterson S, Wu G, Goryanin I, Kell DB, Sander C, Sauro H, Snoep JL, Kohn K, Kitano H. The Systems Biology Graphical Notation. Nature Biotechnology 27: 735-741. (2009) Systems Biology Graphical Notation project: http://www.sbgn.org/.
5. Kohn, K.W. Molecular interaction map of the mammalian cell cycle control and DNA repair systems. *Mol Biol Cell* **10**, 2703-2734 (1999).
6. Kohn, K.W., Aladjem, M.I., Weinstein, J.N. & Pommier, Y. Molecular interaction maps of bioregulatory networks: a general rubric for systems biology. *Mol Biol Cell* **17**, 1-13 (2006).
7. Freeman, T.C., Goldovsky, L., Brosch, M., van Dongen, S., Mazière, P., Grocock, R,J,, Freilich, S., Thornton, J. & Enright A.J. Construction, visualisation, and clustering of transcription networks from microarray expression data. *PLoS Comput Biol.* **3**:2032-42 (2007).
8. Network Visualisation and Analysis of Gene Expression Data using BioLayout *Express*^{3D}. Theocharidis, A., van Dongen, S., Enright A.J. & Freeman, T.C. *Nature Protocols* in press (2009).

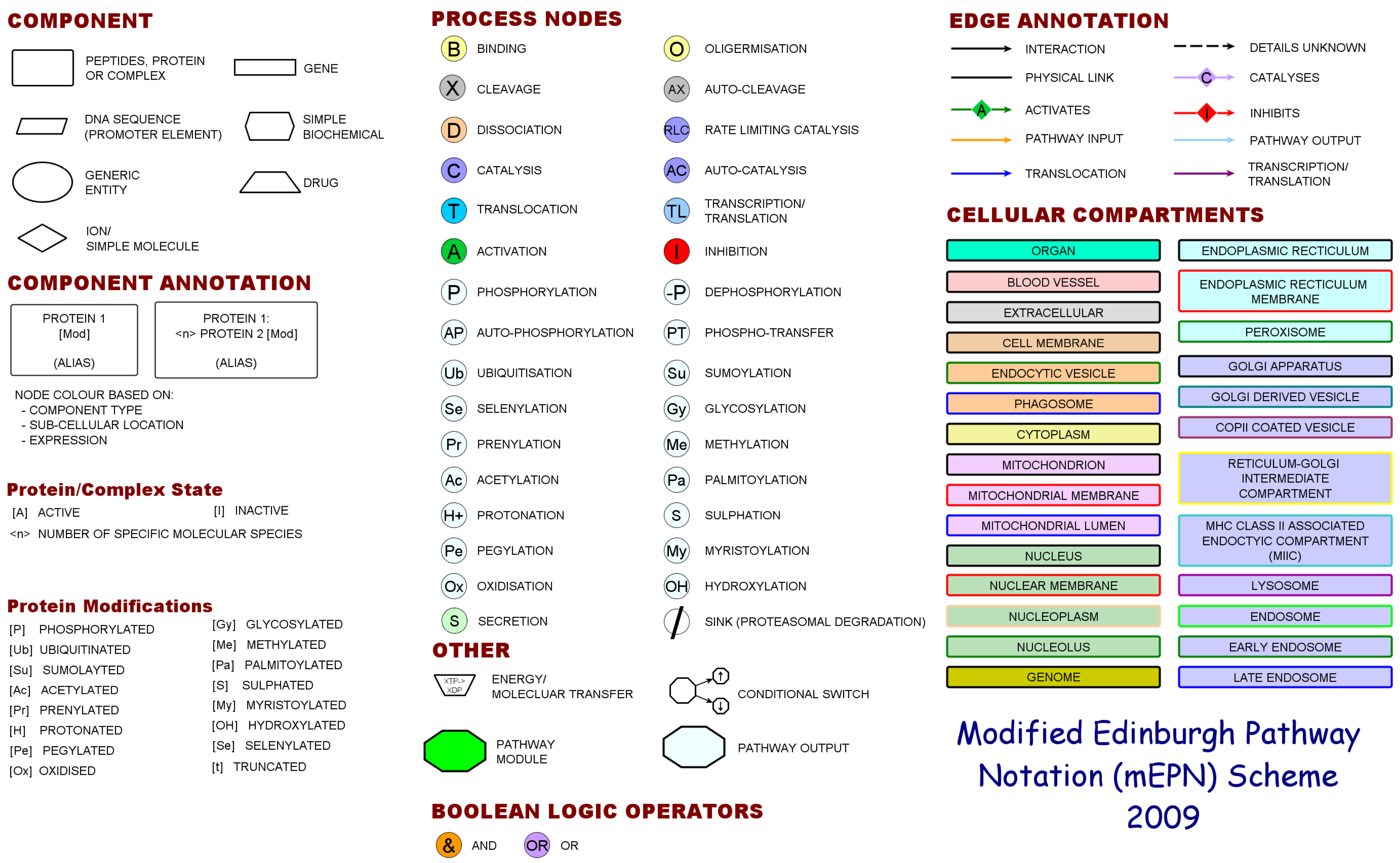

Appendix Figure 1: The modified Edinburgh Pathway Notation (mEPN) scheme 2009. A current list of the notation symbols used for pathway construction. The notation scheme essentially consists of the following categories; components, compartments, Boolean logic operators, edge annotations, process nodes and other nodes necessary to describe pathway components and the relationships between them. Components consist of any interacting species from proteins, complexes, genes, DNA sequence, drug, ion, or other molecular species (pathogens, DNA, RNA). Protein and gene components are annotated using standard gene nomenclature e.g. HUGO or MGD gene symbol with an option to include another common name should one exist. Other annotations such as protein state and modification are added if known. Boolean logic operators (AND, OR) are essential for capturing the dependencies of an interaction. Process nodes provide information as to the nature of the interaction (such as cleavage, translocation, phosphorylation). To date we have identified 32 possible process nodes. Edges are directional and can be coloured for visual impact. Edges carrying specific information about the nature of the interaction with another component or process are annotated with an in-line edge annotation node as a visual representation as to the meaning of the edge. Cellular compartmental information is provided by physical location and backdrop or by colouring nodes according to their sub-cellular location. Unique shapes and textual node annotation are used to distinguish between each element of the notation allowing its interpretation even in the absence of colour. However, colour is a powerful visual aid and can therefore be used for aesthetic purposes and to ease identification of nodes.

Interacting Partner 1				Interacting Partner 2				Interaction Type	Interaction Location	NCBI-PubMed ID
Official Gene Symbol	Gene ID	Interactant Type	Interactant as on map	Official Gene Symbol	Gene ID	Interactant Type	Interactant as on map			
ATM	472	Protein	ATM[P]	IKBKG	4214	Protein	IKBKG[SU]	Binding	nucleus	16497931
ATM	472	Protein	ATM[P]	IKBKG	4214	Protein	IKBKG[SU]	Binding	nucleus	16965765
ATM	472	Protein	ATM[P]	MAP3K?		Protein	MAP3K?	Phosphorylation	nucleus	16410802
ATM	472	Protein	ATM[P]	TP53	7157	Protein	TP53	Phosphorylation	nucleus	9407038
ATM:ATM	472:472	Complex	ATM[P]:ATM[P]					Dissociation	nucleus	16622404
ATM:CHUK:IKBKB:IKBKG	472:1147:3551:4214:23085	Complex	ATM[P]:CHUK:IKBKB:IKBKG[P][Ub]:ERC1(ELKS)	NFKBIA	4792	Complex	NFKB1(p50):RELA:NFKBIA	Phosphorylation	cytoplasm	16965765
ATM:IKBKG	472:4214	Complex	ATM[P]:IKBKG[P][Ub]	CHUK	1147	Protein	CHUK	Binding	cytoplasm	16497931
ATM:IKBKG	472:4214	Complex	ATM[P]:IKBKG[P][Ub]	ERC1	23085	Protein	ERC1(ELKS)	Binding	cytoplasm	15218148
ATM:IKBKG	472:4214	Complex	ATM[P]:IKBKG[P][Ub]	ERC1	23085	Protein	ERC1(ELKS)	Binding	cytoplasm	16497931
ATM:IKBKG	472:4214	Complex	ATM[P]:IKBKG[P][Ub]	IKBKB	3551	Protein	IKBKB	Binding	cytoplasm	16497931
ATM:IKBKG	472:4214	Complex	ATM[P]:IKBKG[P][Ub]					Translocation	nucleustocytoplasm	16965765
BCL2	596	Gene	BCL2	NFKB1(p50):NFKB1(p50)	N/A	Complex	NFKB1(p50):NFKB1(p50)	Activation	nucleus	14668329
BCL2	596	Gene	BCL2	NFKB1(p50):NFKB1(p50)	N/A	Complex	NFKB1(p50):NFKB1(p50)	Activation	nucleus	11567031
BCL2L1	598	Gene	BCL2L1	NFKB1(p50):RELB	N/A	Complex	NFKB1(p50):RELB	Activation	nucleus	17561400
BCL3	602	Protein	BCL3	NFKB1(p50):NFKB1(p50)	N/A	Complex	NFKB1(p50):NFKB1(p50)	Binding	nucleus	17561400
BCL3	602	Protein	BCL3	NFKB1(p50):NFKB1(p50)	N/A	Complex	NFKB1(p50):NFKB1(p50)	Binding	nucleus	16513645
BCL3	602	Protein	BCL3	NFKB1(p50):NFKB1(p50)	N/A	Complex	NFKB1(p50):NFKB1(p50)	Binding	nucleus	11931770
BCL3	602	Protein	BCL3	NFKB2(p52):NFKB2(p52)	N/A	Complex	NFKB2(p52):NFKB2(p52)	Binding	nucleus	15371334
BCL3	602	Protein	BCL3	NFKB2(p52):NFKB2(p52)	N/A	Complex	NFKB2(p52):NFKB2(p52)	Binding	nucleus	17072322
BCL3	602	Protein	BCL3	NFKB2(p52):NFKB2(p52)	N/A	Complex	NFKB2(p52):NFKB2(p52)	Binding	nucleus	15465827
BCL3	602	Protein	BCL3	NFKB2(p52):NFKB2(p52)	N/A	Complex	NFKB2(p52):NFKB2(p52)	Binding	nucleus	14668329
BCL3	602	Protein	BCL3	NFKB2(p52):NFKB2(p52)	N/A	Complex	NFKB2(p52):NFKB2(p52)	Binding	nucleus	16513645
BCL3	602	Protein	BCL3	NFKB2(p52):NFKB2(p52)	N/A	Complex	NFKB2(p52):NFKB2(p52)	Binding	nucleus	9405619
BCL3	602	Protein	BCL3	NFKB2(p52):NFKB2(p52)	N/A	Complex	NFKB2(p52):NFKB2(p52)	Binding	nucleus	17349209
BCL3	602	Protein	BCL3	NFKB2(p52):NFKB2(p52)	N/A	Complex	NFKB2(p52):NFKB2(p52)	Binding	nucleus	10453354
BIRC3	330	Gene	BIRC3	NCOR2(SMRT):NFKB1(p50):RELA(p65)	N/A	Complex	NCOR2(SMRT):NFKB1(p50):RELA(p65)	Activation	nucleus	16846590
BIRC3	330	Gene	BIRC3	NCOR2(SMRT):NFKB1(p50):RELA(p65)	N/A	Complex	NCOR2(SMRT):NFKB1(p50):RELA(p65)	Activation	nucleus	16382138
BIRC3	330	Gene	BIRC3	NFKB1(p50):NFKB1(p50)	N/A	Complex	NFKB1(p50):NFKB1(p50)	Inhibition	nucleus	16846590
BIRC3	330	Gene	BIRC3	NFKB1(p50):NFKB1(p50)	N/A	Complex	NFKB1(p50):NFKB1(p50)	Inhibition	nucleus	16382138
CCL19	6363	Gene	CCL19	NFKB2(p52):RELB	N/A	Complex	NFKB2(p52):RELB	Activation	nucleus	17561400
CCL2	6347	Gene	CCL2(MCP-1)	NFKB1(p50):RELA(p65)	N/A	Complex	NFKB1(p50):RELA(p65)	Activation	nucleus	17561400
CCL5	6352	Gene	CCL5	NFKB1(p50):RELA(p65)	N/A	Complex	NFKB1(p50):RELA(p65)	Activation	nucleus	15117956
CDC37	11140	Protein	CDC37	HSP90AA1	3320	Protein	HSP90AA1	Binding	cytoplasm	15371334
CDC37	11140	Protein	CDC37	HSP90AA1	3320	Protein	HSP90AA1	Binding	cytoplasm	17072322
CDC37	11140	Protein	CDC37	HSP90AA1	3320	Protein	HSP90AA1	Binding	cytoplasm	17072322
CDC37	11140	Protein	CDC37	HSP90AA1	3320	Protein	HSP90AA1	Binding	cytoplasm	11864612
CDC37:HSP90AA1	N/A	Complex	CDC37:HSP90AA1	IKBKB:IKBKG:CHUK:ERC1	N/A	Complex	IKBKB:IKBKG:CHUK:ERC1	Binding		17072322
CDC37:HSP90AA1	N/A	Complex	CDC37:HSP90AA1	IKBKB:IKBKG:CHUK:ERC1	N/A	Complex	IKBKB:IKBKG:CHUK:ERC1	Binding		16970925
CHUK	1147	Protein	CHUK	CHUK	1147	Protein	CHUK	Binding	cytoplasm	15145317
CHUK	1147	Protein	CHUK	IKBKG	8517	Protein	IKBKG	Binding	cytoplasm	11865184
CHUK	1147	Protein	CHUK	IKBKG	8517	Protein	IKBKG	Binding	cytoplasm	12612076
CHUK	1147	Protein	CHUK	IKBKG	8517	Protein	IKBKG	Binding	cytoplasm	10837071
CHUK	1147	Protein	CHUK	NFKB1:NFKB1:HDAC3:NCOR2	4790:4790:8841:9612	Complex	NFKB1(p50):NFKB1(p50):HDAC3:NCOR2(SMRT):[?DNAseq.]	Phosphorylation	nucleus	15494311
CHUK	1147	Protein	CHUK	NFKB1:NFKB1:HDAC3:NCOR2	4790:4790:8841:9612	Complex	NFKB1(p50):NFKB1(p50):HDAC3:NCOR2(SMRT):[?DNAseq.]	Phosphorylation	nucleus	16382138
CHUK	1147	Protein	CHUK	NFKB1:RELA:NCOR2	4790:5970:9612	Complex	NFKB1(p50):RELA:NCOR2(SMRT):[?DNAseq.]	Phosphorylation	nucleus	15494311

Appendix Figure 2: Table of Interaction Data. For each interaction depicted on a pathway diagram it is crucial to keep a record of the supporting evidence for that interaction and unambiguous identifications for each interacting components. As the very minimum it is advisable to store the following information; Official Gene Symbol for both interactants, Gene IDs, the type of interaction, the appearance of the interactant as shown on the map (i.e. if a protein is interacting whilst it is in complex with other proteins then it's full complex name/ details are shown), the type of interaction (usually corresponding to the process node involved), the location of the interaction and the PubMed-ID references for each interaction. Some interactions have multiple sources of references shown on the line below so new interactions are separated by a yellow line break. Other information, such as the technique used to identify the interaction, the cell type in which the interaction was identified and other supporting information can also be stored.

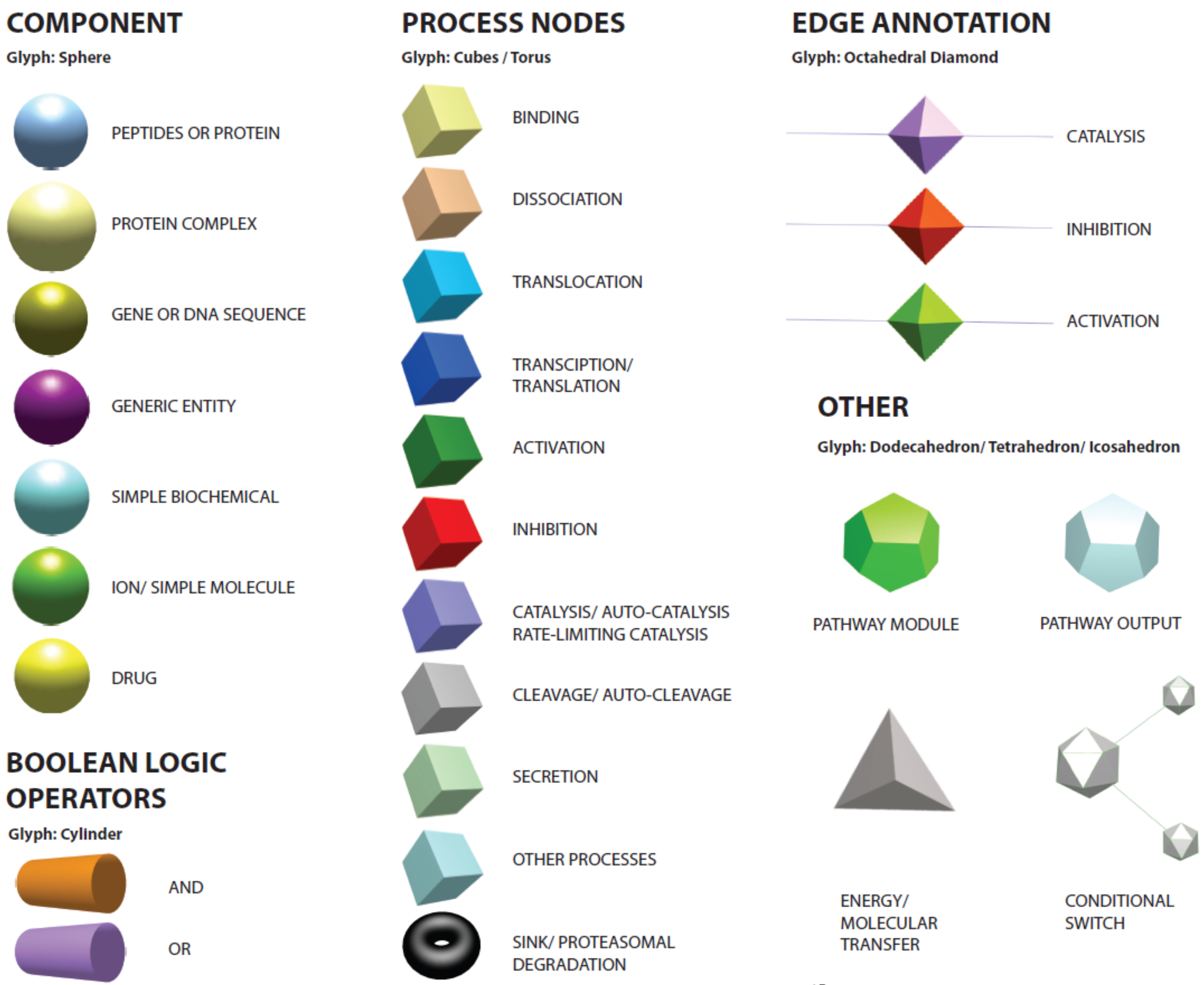

Appendix Figure 3: mEPN 3D Scheme for Process Diagrams as supported by BioLayout *Express*^{3D} (http://www.biolayout.org/) in 3D mode. All components are represented as simple spheres, and can be sized to reflect their complexity/membership e.g. protein complexes can be displayed as larger spheres than nodes representing single proteins. Process nodes and Boolean logic gates are represented as cubes (with the exception of the sink for which we have used a torus), and edge annotations are diamonds. Pathway modules and outputs are shown as dodecahedrons, energy/molecular transfer reactions as tetrahedrons and conditional gates as 3 (one large, two small) icosahedrons. The colour scheme shown reflects that used for the 2D mEPN scheme. All node labels can be rendered visible when viewing the network in the graph visualisation tool BioLayout *Express*^{3D}.
