# Supplementary material for: The mEPN scheme: an intuitive and flexible graphical system for rendering biological pathways
Source: BMC Syst Biol. 2010 May 17;4:65. doi: 10.1186/1752-0509-4-65 (PMC2878301; doi:10.1186/1752-0509-4-65)
Supplement: Additional file 3 — Simple mEPN Worked Examples. Some simple examples of mEPN notation use. [file 1752-0509-4-65-S3.PDF]

mEPN Simple Worked Examples

Binding/ Association

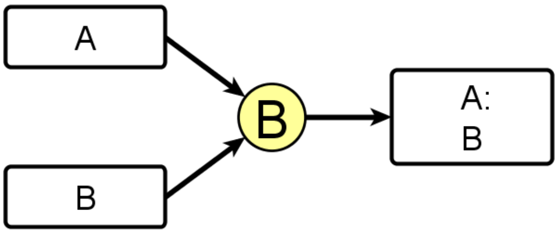

Multiple inputs into a BIND gate are permitted but only one output may arise.

Dissociation

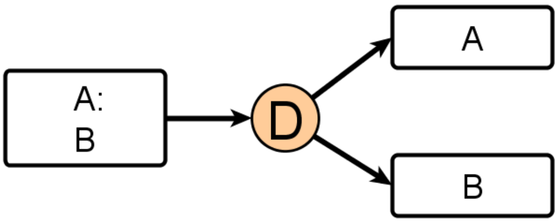

Only one input may enter the DISSOCIATE gate but multiple outputs may leave the gate.

Oligomerisation

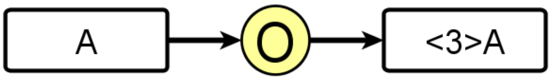

Since oligomerisation is specific to components which associate to themselves only one input is required into the OLIGOMERISE gate and only one output may arise. The number of components required to make the output product is indicated by the annotation in brackets < n > preceding the component name.

Translocation

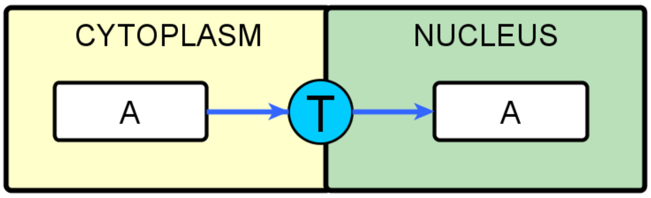

Only one input may enter the TRANSLOCATE gate and only one output may leave the gate. If a component can translocate to several sub-cellular-compartments then this must be shown using separate TRANSLOCATE gates each time.

Sink (proteasomal degradation)

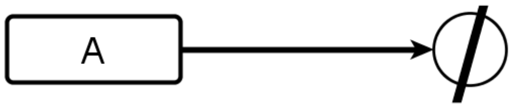

A SINK gate may only have one input entering the gate but no outputs leaving the gate since this gate denotes the removal of a component from the system.

Process (e.g. Phosphorylation)

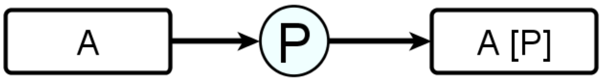

All other process nodes may only have one input entering and one output leaving the process node. However annotated edges may also point to the process node.

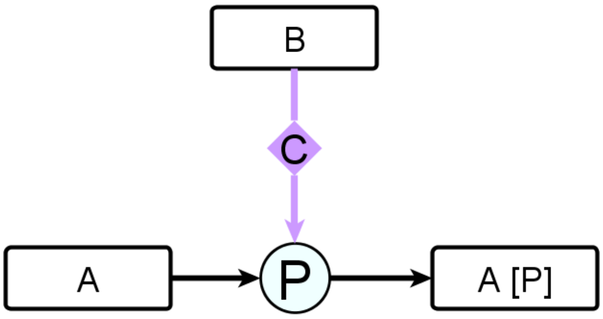

All process nodes may only have one type of the same annotated edge (e.g. activation/ inhibition/ catalysis) pointing to that process.

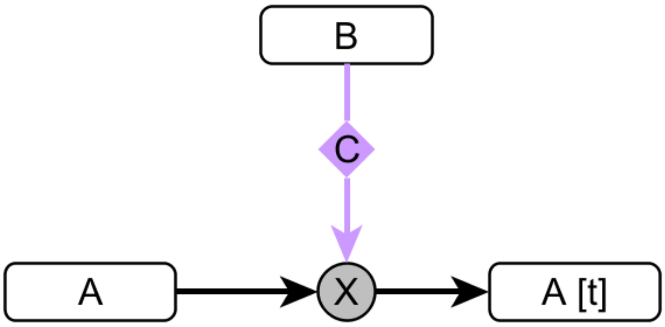

Boolean Logic

OR

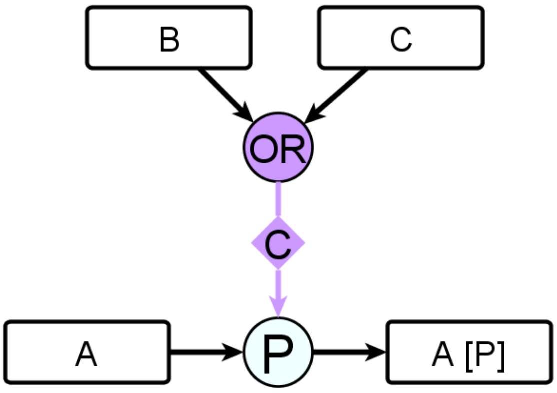

When multiple components can alter the same process independently of each other then an OR gate is used, such that those components input to an OR gate which is connected to the process by the annotated edge.

& (AND)

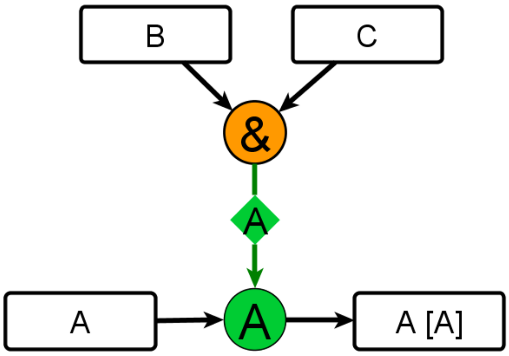

When two or more components are required to alter a process then an & (AND) gate is used to connect them. The & gate is then connected to the process using the appropriate annotated edge.
