# Supplementary material for: The mEPN scheme: an intuitive and flexible graphical system for rendering biological pathways
Source: BMC Syst Biol. 2010 May 17;4:65. doi: 10.1186/1752-0509-4-65 (PMC2878301; doi:10.1186/1752-0509-4-65)
Supplement: Additional file 4 — Comparison of mEPN to SGBN. Comparison of mEPN and SGBN schemes. [file 1752-0509-4-65-S4.PDF]

Comparison of the mEPN Scheme with the SBGN Level 1 (v1.1) specification for the Depiction of Process Diagrams

The mEPN (modified Edinburgh Pathway Notation) and SBGN (Systems Biology Graphical Notation) schemes provide two similar but different ways to depict process diagrams. Each scheme is divided into a set of glyphs to depict different concepts (components, processes, relationships, cellular compartments) for the use of depicting what is known about a biological pathway as a network diagram. Both schemes were developed over roughly the same period of time the SBGN scheme by members of the SBGN community; the mEPN scheme at the Division of Pathway Medicine and Roslin Institute, University of Edinburgh. Both schemes also aspire to fulfil many of the same goals:

1. Allow the detailed representation of diverse biological entities, interactions and pathway concepts
2. Provide a system for presenting pathway knowledge in a semantically and visually unambiguous manner
3. Have network semantics that are sufficiently well defined that software tools can convert graphical models into formal models, suitable for analysis and simulation
4. Be as simple as possible to read and use
5. Understandable to a biologist

We have developed the mEPN scheme so that it is supported by general network editing and visualisation software e.g. yEd graph editor, Cytoscape, BioLayout *Express*^{3D} and therefore the scheme does not require the use of dedicated pathway editing tools. Furthermore, the evolution of the scheme has been driven by its use in depicting a wide range of biological systems.

Although many of the concepts are named differently and many glyphs differ between the two notation systems they are similar enough to compare. Below is a comparison of the main features of both notation schemes and the glyphs used within them. Since different naming conventions are used by the notation systems to describe glyphs, the most similar or conceptually equivalent nodes are compared below. Where a glyph exists in one notation scheme but not in the other a blank space can be found in the adjacent area of the table.

For details of the mEPN scheme see (Freeman et al., 2010) and the SGBN scheme (Le Novère, N. et al., The Systems Biology Graphical Notation. Nature Biotechnology **27**: 735-741 (2009).

Concept	mEPN Node	mEPN Glyph	SBGN equivalent	SBGN Glyph
Pathway Components[1] (Entity Pool Nodes)	Peptide, protein, protein complex	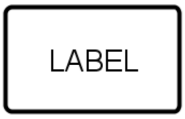	Macromolecule	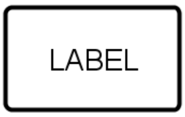
	Gene	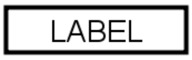	Nucleic acid feature	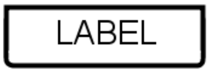
	DNA sequence (e.g. promoter element)	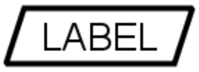		
	Simple biochemical	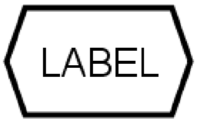	Simple chemical	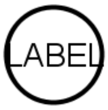
	Ion/ simple molecule	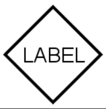	Simple chemical	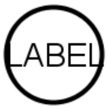
	Generic entity	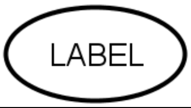	Unspecified entity	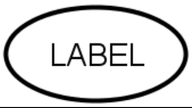
	Drug	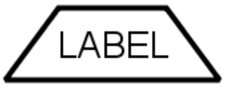		
	Multimers (also complex)	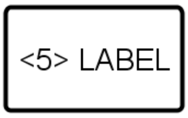	Multimers	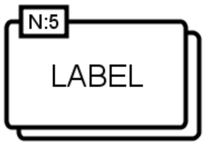
Other	Pathway output[2]	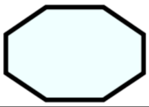	Observable	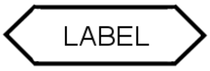
	Pathway module	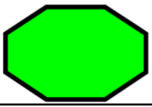	Submap	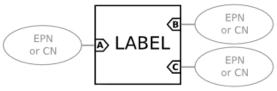
	Energy/ molecular transfer[3]	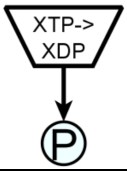		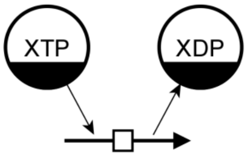
	Conditional switch	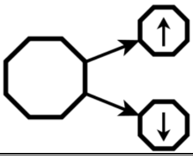		

			**Tag**	LABEL
			**Perturbation**	LABEL
**Cellular Compartment**[4]	**Cellular Compartment**	LABEL COMPARTMENT	**Container Node**	e:INFO LABEL
**Process Nodes (Transitions)**	**Binding**	B	**Association**	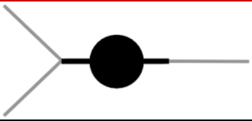
	**Dissociation**	D	**Dissociation**	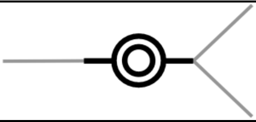
	**Sink**[5]	∅	**Source/Sink**	∅
	**Other processes**[6]	A, I, Sec, O, X, AX, C, AC, Ub, T, TL, Su, P, AP, -P, At, PT, Pe, H+, OH, Ox, Me, Gy, Pa, S, My, Se, Pr	**Transition**	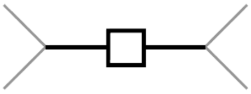
			**Omitted process**	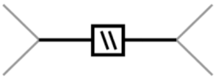
			**Uncertain process**	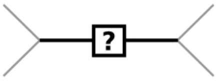

**Boolean Logic Operators**	**AND**	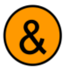	**AND**	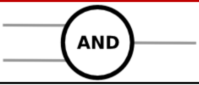
	**OR**	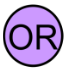	**OR**	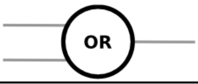
			**NOT**[7]	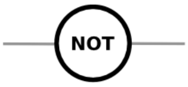
**Interaction Edges (Connecting Arcs)**[8]	**Catalyses**	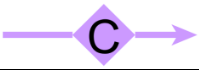	**Catalysis**	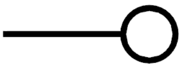
	**Inhibits**	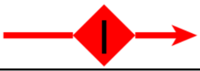	**Inhibition**	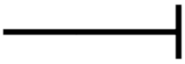
	**Activates**	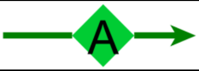	**Stimulation**	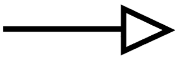
	**Details unknown**	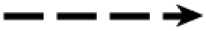		
	**Non-covalent or covalent bond**			
			**Consumption**	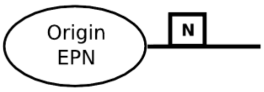
			**Production**	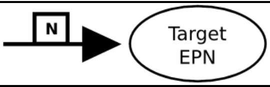
			**Modulation**	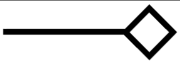
			**Trigger**	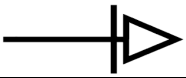
			**Logic Arc**	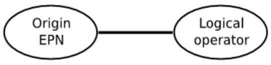
			**Equivalence Arc**	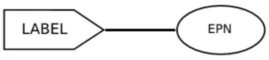
**Component Annotation**[9] **(Auxiliary Items)**	**Component ID**	***Recommended standard gene ID***	**Component ID**	***No standard specified***
	**Component Modifications (examples)**	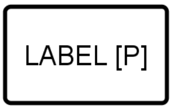	**State Variables (examples)**	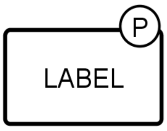
		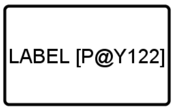		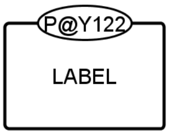

	**Component State**	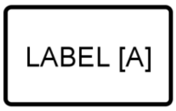	**Unit of information**	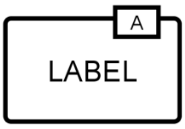

[1] **Pathway Interactant Depiction**

Nodes which are the biological constituents participating in a particular metabolic or signalling pathway are referred to as **Components** in the mEPN and **Entity Pool Nodes** are the equivalent for the SBGN.

[2] **Comparisons of *observable* and *pathway output* glyphs**

Observable and *pathway output* are comparable nodes; *observable* is used to describe a process affected by, or a phenotype generated as a result of pathway signalling. *Pathway Output* is also used for this purpose and always at the end of a series of directional interactions to highlight the consequence of a set of interactions.

[3] **Energy/Phospho Transfer Depiction**

SBGN use two nodes (*simple chemical)* each time to show the transfer of x-tri-phosphate → x-di-phosphate (e.g. ATP → ADP, GTP → GDP, whereas in the mEPN we have chosen to depict these reactions in one glyph which points to the process requiring energy/phosphate transfer. The use of a single glyph to depict energy/phospho-transfer was determined to be the most space efficient way to depict these co-reactions due their widespread occurrence in biology.

[4] **Compartmentalisation**

In both notation schemes the glyphs used to contain nodes present in a given sub-cellular-compartment can take any geometry. The SBGN *container* nodes are also used for containing a *complex* or a *submap* (a node used to encapsulate processes within one glyph).

[5] **Sink Nodes**

Sink is an entity pool node in the SBGN and considered a process in mEPN. Furthermore in mEPN the use of the *sink* node is restricted to defining the removal of a component from a system which in all cases to date has been by proteasomal degradation.

[6] **Other Processes**

Other processes in the mEPN shown reading from left to right in the table are Activation (A), Inhibition (I), Oligomerisation (O), Cleavage (X), Auto-cleavage (AX), Catalysis (C), Auto-catalysis (AC), Translocation (T), Transcription/Translation (TL), Secretion (S), Phosphorylation (P), De-phosphorylation (-P), Auto-phosphorylation (AP), Phospho-transfer (PT), Ubiquitination (Ub), sumoylation (Su), selenylation (Se), glycosylation (Gy), prenylation (Pr), methylation (Me), acetylation (Ac), palmitoylation (Pa), protonation (H+), sulphatation (S), pegylation (Pe), oxidation (Ox), myristoylation (My), and hydroxylation (OH). Use of colour is optional. The nature of processes (transitions) are not generally defined under the current SBGN specification.

[7] **Boolean Logic Operators**

Both notation systems make use of Boolean logic commands AND / OR, however extensive use of the mEPN has yet to find use of the NOT command for signalling pathways and is therefore currently not included from the mEPN notation. Something NOT doing something would seem to be obvious by its omission.

[8] **Edges**

The lines connecting components and process nodes are referred to as **edges** in mEPN or **connecting arcs** in SBGN. The mEPN does not make use of different styles of arrowheads to depict the nature of interactions (edges) instead where appropriate an diamond-shaped inline annotation node carrying a visual clue (a letter symbolising the meaning of the edge e.g. A for activation, I for inhibition) is used to depict the meaning of edges.

[9] **Node Annotations**

No naming conventions are currently recommended by SBGN. When non-standard nomenclature is adopted it frequently leads to ambiguity as to the exact identity as to what is being depicted as multiple component names are often available to describe a given component. Under mEPN we recommend the use of standard nomenclature systems for components e.g. HGNC or MGD conventions to name human or mouse genes/proteins, respectively. Use of standard nomenclature also assists in the comparison and overlay of experimental data with pathway models. Additional information about a component (modifications, states, numbers of given components within a complex) are referred to as **annotations** in the mEPN and **auxiliary items** according to the SBGN.

Other differences

Cloning concept of SBGN

If a component (entity pool node) is duplicated on the map it is indicated by using a 'clone marker' (shading in the bottom third of the node) the purpose of this being to allow the reader a visual indication that the node has been duplicated elsewhere on the map. Whilst on a map of moderate size this maybe practical (allowing the reader to identify how many times the node is duplicated) on a larger scale and where multiple nodes are cloned it may become difficult to trace how many times the node is cloned.

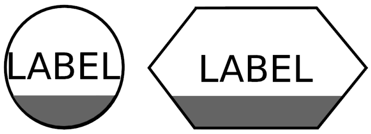

In contrast to the SBGN, mEPN usage rules dictate that a component node (proteins, complexes, etc) representing a given entity may only be represented once in the in a given sub-cellular compartment (this rule does not apply to ubiquitous components or reactions e.g. simple ions, energy transfer reaction nodes). The trade off here is that so called 'hub' nodes (those with many connections) will have many edges emanating from them to other components in various different locations of the map. However, the number of edges leaving each node gives the reader an exact indication of its connections and hence activity in the map without the need for scanning the entire diagram to find cloned nodes. Furthermore, the SBGN has laid out rules as to which

glyphs may be cloned and which then require clone markers, adding yet another set of rules that map constructers must learn. Although both notation systems do not provide a perfect solution to dealing with highly connected nodes we feel the mEPN rule (biological component can be shown only once in a given sub-cellular compartment) is a more practical resolution for readers and constructors of the diagram for the reasons discussed above and also since repetition of identical nodes consumes more space on the map.
